# *Fbw7* Repression by Hes5 Creates a Feedback Loop That Modulates Notch-Mediated Intestinal and Neural Stem Cell Fate Decisions

**DOI:** 10.1371/journal.pbio.1001586

**Published:** 2013-06-11

**Authors:** Rocio Sancho, Sophia M. Blake, Christian Tendeng, Bruce E. Clurman, Julian Lewis, Axel Behrens

**Affiliations:** 1 Mammalian Genetics Laboratory, CR UK London Research Institute, Lincoln's Inn Fields Laboratories, London, United Kingdom; 2 Vertebrate Development Laboratory, CR UK London Research Institute, Lincoln's Inn Fields Laboratories, London, United Kingdom; 3 University of Washington School of Medicine, Seattle, Washington, United States of America; Washington University, United States of America

## Abstract

FBW7 is a crucial component of an SCF-type E3 ubiquitin ligase, which mediates degradation of an array of different target proteins. The *Fbw7* locus comprises three different isoforms, each with its own promoter and each suspected to have a distinct set of substrates. Most FBW7 targets have important functions in developmental processes and oncogenesis, including Notch proteins, which are functionally important substrates of SCF(Fbw7). Notch signalling controls a plethora of cell differentiation decisions in a wide range of species. A prominent role of this signalling pathway is that of mediating lateral inhibition, a process where exchange of signals that repress Notch ligand production amplifies initial differences in Notch activation levels between neighbouring cells, resulting in unequal cell differentiation decisions. Here we show that the downstream Notch signalling effector HES5 directly represses transcription of the E3 ligase *Fbw7β*, thereby directly bearing on the process of lateral inhibition. *Fbw7* ^Δ/+^ heterozygous mice showed haploinsufficiency for Notch degradation causing impaired intestinal progenitor cell and neural stem cell differentiation. Notably, concomitant inactivation of *Hes5* rescued both phenotypes and restored normal stem cell differentiation potential. In silico modelling suggests that the NICD/HES5/FBW7β positive feedback loop underlies *Fbw7* haploinsufficiency. Thus repression of *Fbw7β* transcription by Notch signalling is an essential mechanism that is coupled to and required for the correct specification of cell fates induced by lateral inhibition.

## Abstract 2

A novel intracellular positive feedback loop connects Fbw7 and Notch: while Fbw7 down-regulates the stability of NICD protein, it is also itself transcriptionally down-regulated by NICD target Hes5.

## Author Summary

The Notch signalling pathway is a highly conserved system that controls cell differentiation decisions in a wide range of animal species and cell types, and at different steps during cell lineage progression. An important function of the Notch pathway is in lateral inhibition—an interaction between equal adjacent cells that drives them towards different final states. The basic principle of lateral inhibition is that activation of the Notch cell surface receptor represses production of the Notch ligand (also borne on the cell surface). Consequently, cells expressing less Notch produce more Notch ligand that can activate the Notch pathway in neighboring cells and thereby amplify the differences between these cells. However, the additional regulatory circuits required to fine-tune this delicate process have so far remained elusive. Here we describe the identification of a novel intracellular positive feedback loop that connects Fbw7 (the ubiquitin ligase responsible for targeting Notch for degradation) and Notch itself. We show that Fbw7 reduces the stability of Notch intracellular domain (NICD) protein, as previously established, but also that the *fbw7* gene is itself transcriptionally downregulated by the Notch effector Hes5. Thus we conclude that increased Notch activity causes NICD stabilisation. Further, we demonstrate that perturbation of this regulatory loop is responsible for the Fbw7 haploinsufficiency observed for Notch-dependent functions in intestine and brain stem cells.

## Introduction

FBW7 belongs to the family of SCF (Skp1, Cul1, F-box)-E3 ligases, which degrades several oncoproteins that function in cellular growth and division pathways, including c-MYC, CYCLIN-E, c-JUN, and Notch proteins. Three FBW7 isoforms have been identified (FBW7α, FBW7β, FBW7γ), each with an isoform-specific first exon, linked to 10 shared exons. Each isoform is expressed from its own promoter allowing isoform-specific transcriptional regulation and tissue-specific expression. Whether FBW7 isoforms show preferential degradation of substrates is still controversial, although studies have shown that c-MYC, CYCLIN-E, and PIN1 are degraded specifically by FBW7*α* [1]–[3]. FBW7β, however, has remained more enigmatic, partly due to its lower absolute mRNA abundance in several cell lines and tissues, when compared to *Fbw7α* [2],[4]. A further level of complexity of FBW7 function is added by the fact that different substrates are regulated in a tissue-specific manner by FBW7 [4]–[6].

Intestinal stem cells are located in the crypt base where they produce rapidly proliferating daughter cells, transit amplifying (TA) cells, which fill the crypts and gradually lose their progenitor identity to differentiate into the two main epithelial lineages upon reaching the crypt-villus junction. The absorptive lineage comprises all enterocytes, while the secretory lineage is composed of goblet cells (secreting protective mucins), enteroendocrine cells (secreting hormones like serotonin or secretin), and Paneth cells (secreting bactericidal proteins, and restricted to the bottom of the crypt in the small intestine [7]). TA cells inevitably encounter a binary decision point that will determine whether they differentiate along an absorptive or a secretory pathway [8],[9]. The Notch pathway is a key regulator of this choice. *RBP-J*κ conditional knockout mice or treatment of mice with a γ-secretase inhibitor results in secretory cell expansion [10]. Conversely, in transgenic mice expressing the activated form of Notch1 (NICD1), goblet cells are absent and the proliferative compartment is expanded [11]. FBW7 has proven to be a critical regulator of intestinal stem cell differentiation, as its deletion in the gut significantly increased NICD1 protein levels and reduced goblet cell numbers [5].

Another example demonstrating the importance of FBW7 in Notch biology and function is that of neural stem cells (NSCs). At the beginning of neurogenesis, neuroepithelial stem cells give rise to radial glial stem cells (RGCs), which represent the major population of NSCs at later stages of embryonic cortex development [12]. Notch activity is very high in RGCs, and needs to be downregulated for neuronal differentiation to occur [13]. Overexpression of NICD1 has been shown to be sufficient to promote radial glial identity during embryogenesis, while abrogation of Notch signalling leads to depletion of RGCs [14],[15]. In line with these observations, we have shown that absence of *Fbw7* in NSCs causes severely impaired RGC stem cell differentiation, accompanied by accumulation of the FBW7 substrate NICD1 [4].

The Notch signalling pathway is a highly conserved pathway that is not only involved in the development and stem cell biology of the mammalian intestine and brain, but controls cell differentiation decisions in a wide range of metazoan species, in a broad range of cell types within a single organism, and at different steps during cell lineage progression.

Mammals have 4 Notch receptors (Notch1–4), 3 Delta-like ligands (Dll1, 3, 4), and 2 Serrate-like ligands termed Jagged (Jagged1 and 2). Ligand binding triggers a complex proteolytic cascade involving ADAM proteases and an intramembranous enzyme complex called γ-secretase, which results in the release of the cytoplasmic domain of Notch proteins from the plasma membrane. The Notch intracellular domain (NICD) shuttles all the way from the cell membrane to the nucleus, where it binds to RBP-Jκ and other proteins, and establishes an activator complex, leading to the expression of target genes. In mammals, the best-characterized Notch target genes belong to the Hes (Hairy Enhancer of Split) and Herp/Hey (Hes-related repressor proteins with Y-box) family of basic helix-loop-helix (bHLH) transcriptional repressors [16],[17].

An important function of the Notch pathway is in lateral inhibition—an interaction between equal adjacent cells that serves to drive them towards different final states. The basic principle of lateral inhibition is that activation of Notch represses production of the Notch ligand. Consequently, the cell with lower Notch activity produces more ligand, and this activates Notch signalling in the neighbouring cell, which results in reduced ligand production. This in turn enables the cell with lower Notch activity to increase its ligand production even further, because it receives a weakened inhibitory signal back from its neighbours. The effect of this feedback loop is that any initial difference in Notch activity between them, whether stochastic or genetically controlled, is amplified to drive the neighbouring cells into opposite Notch-level status and hence into different developmental pathways [18].

In this manuscript we describe the identification of a novel intracellular positive feedback loop that connects Fbw7 and Notch: FBW7 not only downregulates stability of NICD protein, as previously established, but is also itself transcriptionally downregulated by NICD (via the action of NICD on Hes5). We demonstrate that FBW7 is haploinsufficient for Notch-dependent physiological functions, as *Fbw7* ^Δ/+^ heterozygous mice show impaired differentiation of intestinal goblet cells and NSCs. This haploinsufficiency is greatly dependent on the newly identified negative transcriptional regulation of the *Fbw7β* promoter by Hes5 protein. We can further show for the first time a pronounced isoform-specific function of FBW7β in driving Notch1 intracellular domain (NICD1) degradation. Genetic rescue experiments and computer modelling of Notch signalling suggest that the FBW7β/NICD/HES5 feedback loop modulates Notch-dependent cell fate decisions and underlies Fbw7 haploinsufficiency.

## Results

### Haploinsufficient Fbw7 Function in Intestinal and NSC Fate Decisions

We have previously used conditional gut-specific knock-out mice allowing for deletion of *Fbw7* specifically in the intestinal tissue to investigate Fbw7 function in gut biology and tumourigenesis. Mice harbouring an *Fbw7* allele in which exon5 was flanked by two loxP sites were crossed to *villin-cre* transgenic mice, previously shown to provide efficient gut-specific Cre activity [19]. Deletion of exon 5, which encodes most of the F-box, an essential domain of FBW7, disrupts the *Fbw7* open reading frame and prevents production of detectable FBW7 protein [20]. Mono-allelic *FBW7* mutations are frequently observed in human colorectal cancer (CRC) and we described that also in the mouse *Fbw7* heterozygosity greatly increased intestinal tumour number in the APC^Min/+^ mouse model [5], indicating that FBW7 haploinsufficiency in intestinal tumour formation is conserved between mouse and human.

**Figure 1:**
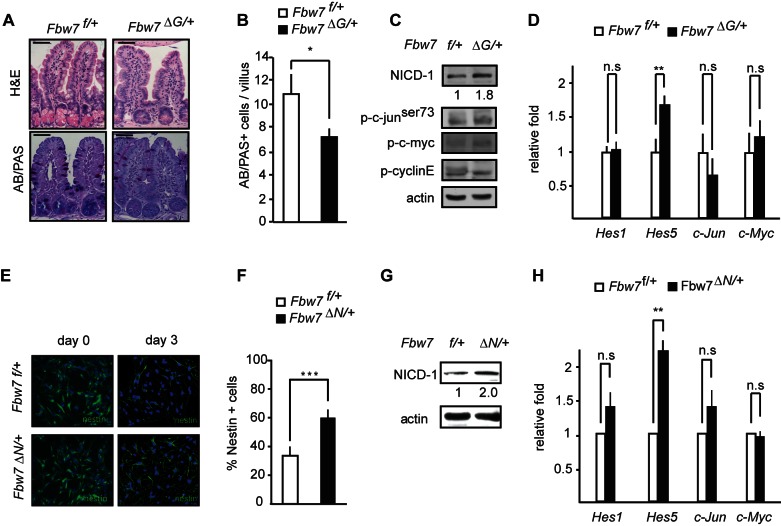
Fbw7 is haploinsufficient for Notch in the gut and NSCs. (a) H&E and AB/PAS staining in intestinal tissue from *Fbw7^f/+^* or *Fbw7^ΔG/+^* mice. (b) Quantification of goblet (AB-PAS+) cells in intestinal tissue from *Fbw7^f/+^* or *Fbw7^ΔG/+^* mice. (c) Western analysis of protein lysates from intestinal cells isolated from *Fbw7^f/+^* or *Fbw7^ΔG/+^* mice (numbers indicate the fold induction of NICD normalized to actin). (d) Q-PCR analysis of *Hes1, Hes5, c-Jun* and *c-Myc* transcripts in *Fbw7^ΔG/+^* intestinal cells compared to *Fbw7^f/+^* (relative fold induction after normalizing to actin ± SEM, *n*≥3 for each genotype). (e) Nestin staining of NSCs isolated from *Fbw7^f/+^* or *Fbw7^ΔN/+^* mice. (f) Quantification of Nestin+ cells in NSCs isolated from *Fbw7^f/+^* or *Fbw7^ΔN/+^* mice (percentage positive cells ± SEM, *n*≥20 for each genotype). (g) Western blot analysis of protein lysates from *Fbw7^f/+^* or *Fbw7^ΔN/+^* NSCs for NICD-1 (numbers indicate the fold induction of NICD normalized to actin). (h) Q-PCR analysis of *Hes1, Hes5, c-Jun* and *c-Myc* transcripts in *Fbw7^ΔN/+^* NSCs compared to *Fbw7^f/+^* NSCs (relative fold induction after normalizing to actin ± SEM, *n*≥3 for each genotype).

*Fbw7^f/+^; villin-cre* heterozygous (*Fbw7^ΔG/+^*) mice showed a significant decrease in goblet cell differentiation, suggesting that FBW7 is a haploinsufficient regulator of goblet cell fate decisions in the gut (Figure 1a,b). FBW7 controls the stability of several proteins with well-documented functions in the intestine such as NICD [21], N-terminally phosphorylated c-JUN [22], c-MYC [1], and CYCLIN-E [23]. We next determined to what extent protein levels of these substrates were deregulated by heterozygous *Fbw7* inactivation. Western blot analysis revealed an increase in NICD1, but the protein levels of N-terminally phosphorylated c-JUN, c-MYC, and CYCLIN-E were less affected in *Fbw7^ΔG/+^* mice (Figure 1c, Figure S1a). To have a more quantitative measure for NOTCH and c-JUN activity, we performed q-PCR analysis of classical target genes of both transcription factors (Figure 1d, Figure S1d–e). In agreement with the western blot analysis, *c-Jun* and *c-Myc* mRNA levels were unaffected in *Fbw7^ΔG/+^* intestines, while *Hes5* mRNA was significantly increased (Figure 1d). Thus only NICD1, but none of the other substrates tested, was increased in *Fbw7* heterozygous mice.

To further investigate FBW7 haploinsufficiency in a second tissue, we analysed NSCs from FBW7 wild-type and heterozygous animals.

We generated conditional brain-specific knock-out mice allowing the deletion of *Fbw7* specifically in the brain. The aforementioned *Fbw7 ^f/+^* mice were crossed to *Nestin:Cre* transgenic mice previously shown to provide efficient brain-specific Cre activity [4].

NSCs were prepared from E13.5 embryos and maintained as an adherent monolayer culture. These cultures were induced to differentiate by withdrawing growth factors, and 3 d after the induction of differentiation, the percentage of remaining Nestin-positive NSCs was determined. A significantly higher number of *Fbw7^ΔN/+^* NSCs retained Nestin expression as compared to the wild-type controls (Figure 1e, 1f), which coincided with elevated NICD1 protein levels in *Fbw7^ΔN/+^* NSCs (Figure 1g, Figure S1b). Consequently, mRNA levels of *Hes5*, but not *c-Jun* or *c-Myc*, were significantly elevated in *Fbw7 ^ΔN/+^* NSCs (Figure 1h, Figure S1c).

Thus Fbw7 is haploinsufficient for Notch degradation during both goblet cell and NSC differentiation.

### Distinct Regulation of the *Fbw7β* Locus

**Figure 2:**
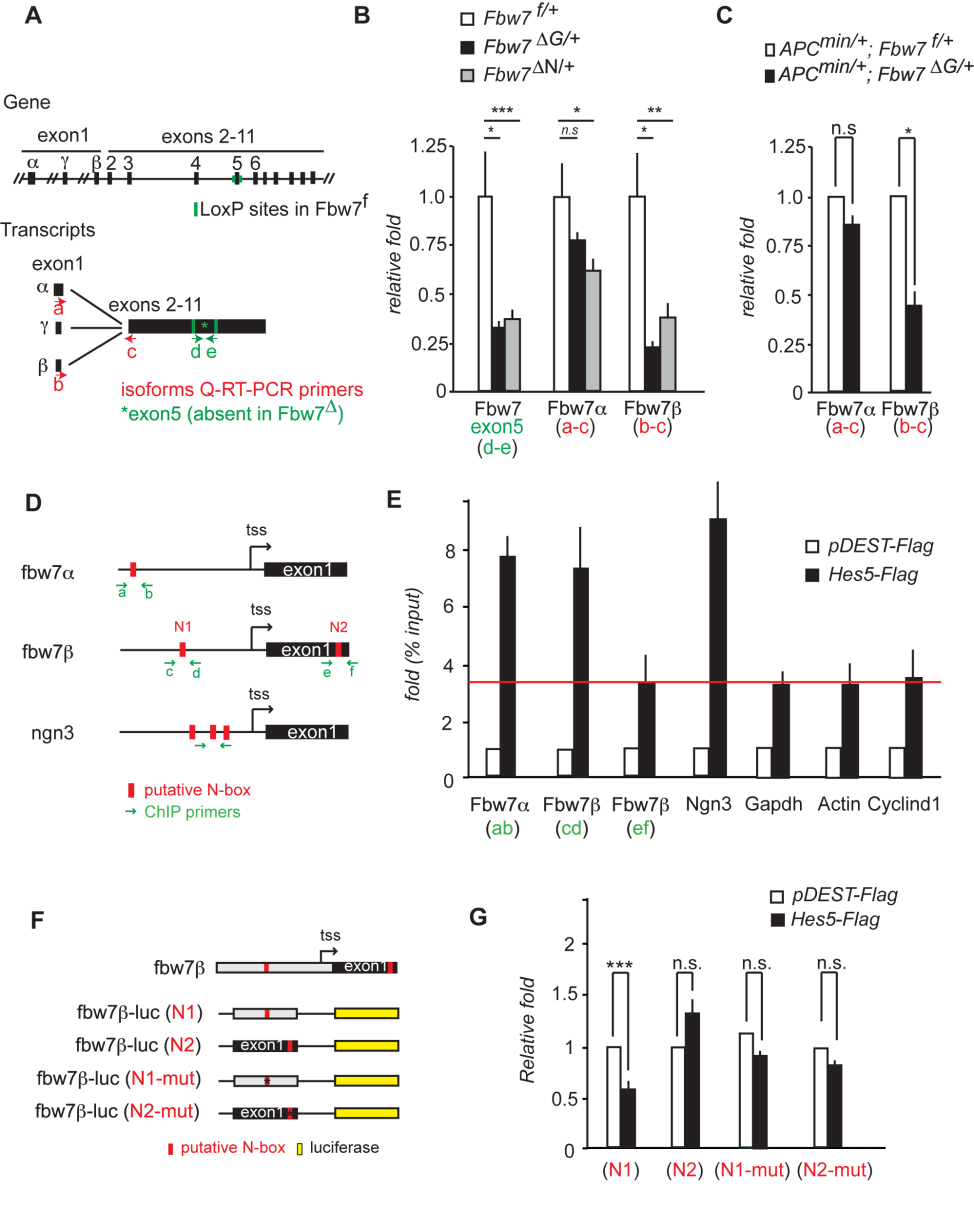
Fbw7*β* is transcriptionally regulated by Hes5. (a) Schematic representation of the *Fbw7* genomic locus (adapted from [24]). mRNA transcripts for the different *Fbw7* isoforms and Q-PCR primers used to detect *Fbw7* isoforms are depicted in the figure. (b) Q-PCR analysis of *Fbw7α*, *Fbw7β*, and *Fbw7*(exon5) in intestinal cells and NSCs isolated from *Fbw7^f/+^* or *Fbw7^ΔG/+^* mice and *Fbw7^f/+^* or *Fbw^ΔN/+^* NSCs, respectively (relative fold induction after normalizing to actin ± SEM, *n*≥3 for each genotype). (c) Q-PCR analysis of *Fbw7α* and *Fbw7β* in intestinal tumours isolated from *APC^min/+^*; *Fbw7^f/+^* or *APC^min/+^*; *Fbw7^ΔG/+^* mice (relative fold induction after normalizing to actin ± SEM, *n*≥3 for each genotype). (d) Schematic representation of *Fbw7α*, *Fbw7β*, and *Ngn3* promoter regions. Red boxes denote consensus N-box sites. Green arrows indicate primers used for ChIP. (e) ChIP was performed using HCT116 cells transfected with p-Dest-flag or p-Dest-Hes5-flag. Flag binding to the consensus sites in *Fbw7α*, *Fbw7β*, and *Ngn3* promoters was determined by Q-PCR. Data were represented as fold activation of percentage input versus the p-DEST-Flag samples. Red line denotes background-binding activity. (f) Schematic representation of the different *Fbw7β*-luciferase constructs generated. Red rectangles represent putative N-boxes. Crossed red rectangles represent mutated N-boxes. (g) HCT116 cells were transfected with *Fbw7β*–N1, *Fbw7β*–N2, *Fbw7β*–N1-mut, pGL3–*Fbw7β*–N2-mut together with p-Dest-Hes5-Flag overexpression vector or p-Dest-flag as a control. Data represent luciferase activity relative to *Fbw7β*–N1+pDest-flag transfected cells.

To understand the haploinsufficiency of FBW7 function, we explored the possibility of feedback regulation and investigated the expression of *Fbw7* in *Fbw7^f/+^* control and *Fbw7^ΔG/+^* heterozygous intestine and *Fbw7^ΔN/+^* heterozygous NSCs. The *Fbw7* locus encodes three different *Fbw7* isoforms (*Fbw7α*, *Fbw7β*, *Fbw7γ*) that are not generated by alternative splicing; rather, each isoform has its unique 5′UTR and is transcribed from an isoform-specific promoter (Figure 2a) [24]. We have previously shown that the α and β *Fbw7* isoforms are expressed in the intestine and the brain, whereas the γ isoform was undetectable [4],[5]. Using quantitative qPCR analysis we show that the *Fbw7α* isoform is 170- and 10-fold more abundant than the *Fbw7β* isoform in the intestine and NSCs, respectively (Figure S2). To circumvent a potential alteration in mRNA stability of the *Fbw7*Δ allele, we used Q-PCR primers located in exon5, which is missing in the *Fbw7*Δ allele (Figure 2a). Thus using this approach exactly 50% of the normal amount of *Fbw7* mRNA is expected in *Fbw7^Δ/+^* heterozygous cells. However, *Fbw7* mRNA levels in *Fbw7^ΔG/+^* intestine and *Fbw7 ^ΔN/+^* NSC were only 30% of controls, a reduction of about 40% from the expected expression of the intact allele (Figure 2b). Q-PCR analysis using isoform-specific primers, which detect both the wild-type and the Δ*Fbw7* alleles (Figure 2a), showed that *Fbw7α* mRNA levels were only slightly reduced in control *Fbw7^f/+^* and *Fbw7^ΔG/+^* intestines as well as in *Fbw7^ΔN/+^* NSCs. In contrast, expression of *Fbw7β* mRNA was greatly reduced in *Fbw7^ΔG/+^* intestines and *Fbw7^ΔN/+^* NSCs (Figure 2b). Mono-allelic (i.e., heterozygous) *FBW7* mutations are frequently observed in human CRC, and *Fbw7* heterozygosity greatly increases intestinal tumour number in the APC^Min/+^ mouse model [5]. Similarly, a reduction in *Fbw7β* mRNA was observed in tumours from APC^Min/+^; *Fbw7^ΔG/+^* mice compared to APC^Min/+^; *Fbw7^f/+^* tumours (Figure 2c).

To gain insights into the mechanism of *Fbw7* transcriptional regulation, we performed an in silico transcription factor binding site analysis of the genomic *Fbw7* locus. This revealed the presence of putative N-box sites, the consensus binding element recognized by HES transcription factors, in the promoters of both *Fbw7α* and *Fbw7β* (Figure 2d). Our attempts to perform Chromatin immunoprecipitation (ChIP) analysis on endogenous HES5 failed as we were unable to identity a suitable Hes5-specific antibody (Figure S3). For this reason Flag-HES5 was overexpressed in HCT116 colon cancer cells and ChIP performed using Flag antibody. This revealed binding of HES5 protein to the N-box in the *neurogenin3* promoter (*NGN3*), a known HES target gene [25], which served as a positive control. However, we also observed some unspecific DNA binding of FLAG-HES5 relative to control vector transfected cells at the *GAPDH*, *β-ACTIN*, and *CYCLIND1* promoters, which all served as negative controls. HES5 bound to predicted N-box elements present in the *FBW7α* and *FBW7β* promoters to a similar extent to *NGN3*, but did not bind significantly to a putative N-box in exon1 of *FBW7β* (Figure 2e).

When inserted into a luciferase reporter construct, the *FBW7β* promoter fragment including the functional (N1) Hes5 binding site (*FBW7β* N1-luc) was repressed by HES5 overexpression, whereas an *FBW7β* fragment covering exon1 (*FBW7β*–N2-luc) and lacking the N1 site was unaffected. Mutation of the N1 N-box (*FBW7β* N1-mut-luc) rendered the *FBW7β* promoter fragment unresponsive to HES5 (Figure 2f,g).

Together these data point very strongly to a specific role for HES5 in regulating *FBW7* transcription.

### Hes5 Represses *Fbw7β* Transcription

**Figure 3:**
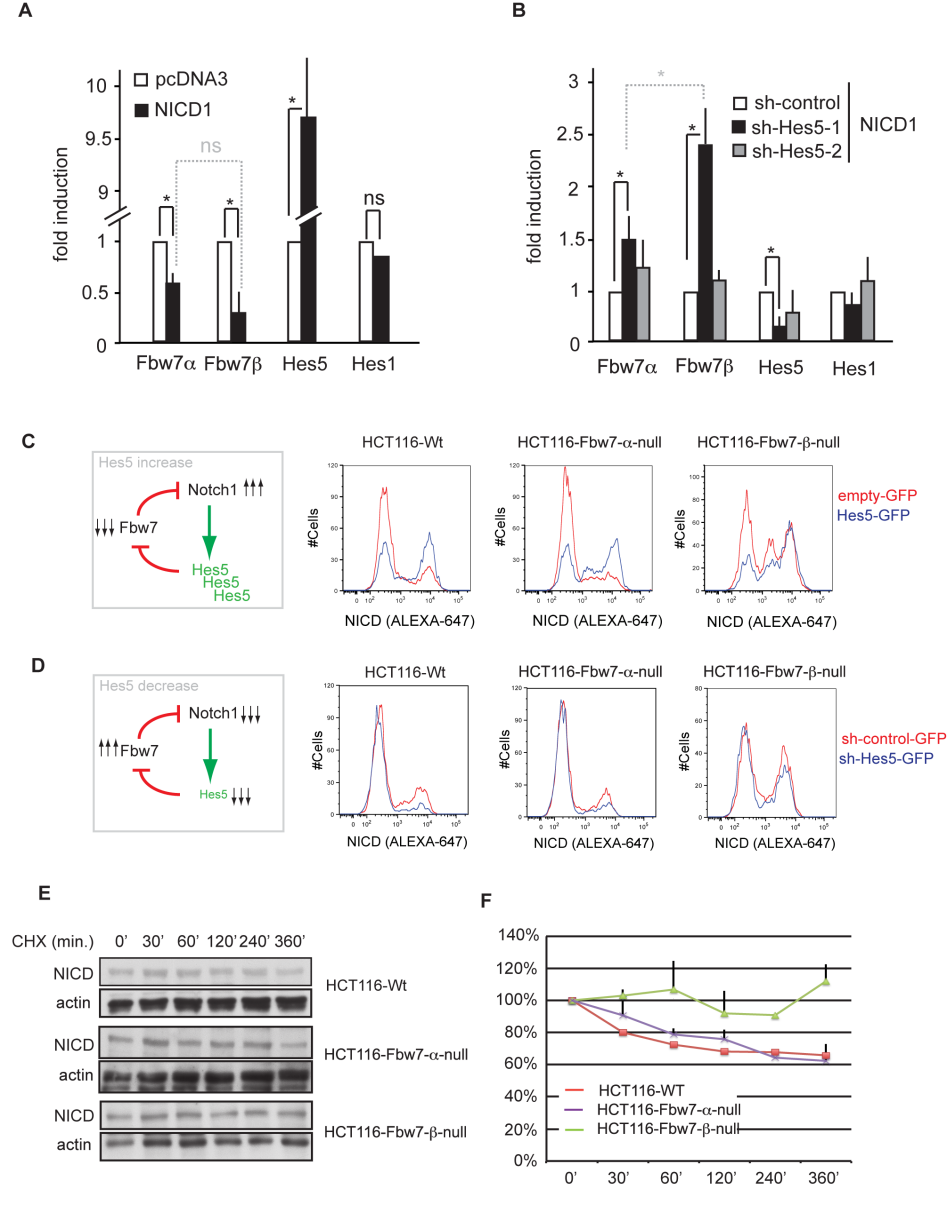
Hes5 represses Fbw7*β* transcription and induces an increase in NICD in a cell autonomous manner. (a) Q-PCR analysis of *Fbw7α*, *Fbw7β*, *Hes5*, and *Hes1* in HCT116 cells transfected with pcDNA3 or pcDNA3-NICD. (b) Q-PCR analysis of *Fbw7α*, *Fbw7β*, *Hes5*, and *Hes1* in HCT116 cells transfected with pcDNA3-NICD in combination with either p-Super-sh-control or p-Super-sh-Hes5-1 and p-Super-sh-Hes5-2 (specific silencers for Hes5). (c) FACS analysis of intracellular NICD in HCT116-Wt, HCT116-Fbw7-α-null, or HCT116-Fbw7-β-null cells transfected with either pCMV6-Gfp or pCMV6-Hes5-gfp plasmid (histograms represent the levels of NICD on GFP+ gated cells). (d) FACS analysis of intracellular NICD in HCT116-Wt, HCT116-Fbw7-α-null, or HCT116-Fbw7-β-null cells transfected with either sh-control-Gfp or sh-Hes5-gfp plasmid (histograms represent the levels of NICD on GFP+ gated cells). (e) Western blot analysis of NICD and ACTIN in HCT116-Wt, HCT116-Fbw7-α-null, or HCT116-Fbw7-β-null cells after treatment with cycloheximide for the indicated time points. (f) Quantification of NICD levels normalized to actin in HCT116-Wt, HCT116-Fbw7-α-null, or HCT116-Fbw7-β-null cells after treatment with cycloheximide for the indicated time points.

To further validate *FBW7* as a direct transcriptional target of HES5, NICD1 was ectopically expressed in HCT116 colon cancer cells. NICD1 expression resulted in increased *HES5* mRNA levels, but had no effect on *HES1*. Moreover, *FBW7β* and to a lesser extent *FBW7α* mRNA levels were strongly repressed (Figure 3a). shRNA-mediated knock-down of *HES5* reversed the repression of *FBW7α* and *FBW7β* expression (Figure 3b). Similar results were obtained in NSCs (Figure S4a,b).

The direct repression of *FBW7β* expression, and to a lesser extent, of *FBW7α*, by HES5 implies that NICD1, HES5, and FBW7 are connected through a feedback loop. This leads to the unexpected prediction that overexpression of HES5 should result in a cell-autonomous increase in NICD1 protein levels (Figure 3c), but that this increase should be impaired in cells deleted for the E3 ligase (that is, FBW7) regulating NICD turnover. To test this hypothesis, we used a set of human colon cancer HCT116 cell lines that have homozygous isoform-specific *FBW7*-null mutations [2].

GFP-tagged-HES5 or GFP alone was overexpressed in HCT116 *FBW7–wt*, *FBW7α-null*, and *FBW7β-null* cells followed by intracellular NICD staining and FACS analysis. FACS analysis on GFP+ gated cells revealed that NICD1 protein levels in HCT116 are not uniform, rather that there are two distinct subpopulations with different NICD1 levels. This resembles the bi-stability observed when lateral inhibition operates, and thus should be affected by the intracellular NICD—>Hes5 —| Fbw7 —| NICD positive feedback loop. In line with this, the majority of *FBW7-wt* cells were in the low-NICD state, while a greater proportion of *FBW7β-null* cells were in the high-NICD state (Figures S5a–c and S6a). Expression of HES5-GFP shifted these proportions, leading to a marked cell-autonomous increase in the percentage of cells in the high-NICD1 state in *FBW7–wt* and *FBW7α-null* cells, but this increase was drastically impaired in *FBW7β*-*null* cells (Figure 3c and Figure S5). Conversely, silencing *HES5* (sh-*HES5*-GFP) led to a cell-autonomous reduction in the percentage of cells in a high-NICD state in *FBW7-wt* and *FBW7α*-*null* cells, which was compromised in *FBW7β*-*null* cells (Figure 3d and Figure S5). These data imply that FBW7β is the predominant isoform involved in the NICD1/HES5/FBW7 feedback loop.

To formally show that FBW7β regulates NICD degradation, we performed cycloheximide chase experiments for NICD turnover in *FBW7-wt*, *FBW7α*-*null*, and *FBW7β*-*null* cells. We found that NICD turnover was reduced in *FBW7β-null* cells by comparison with *FBW7-wt* and *FBW7α*-*null* cells (Figure 3e, 3f). Accordingly, we observed less ubiquitylation of NICD in *FBW7β*-*null* cells (Figure S8c). Q-PCR analysis performed in the same set of Fbw7-mutant cell lines confirmed that only loss of *FBW7β* resulted in increased *HES5* mRNA levels (Figure S6b). Together, these data demonstrate a crucial role of FBW7β in regulation of NICD turnover.

### Fbw7 Haploinsufficiency Requires Hes5 Function

**Figure 4:**
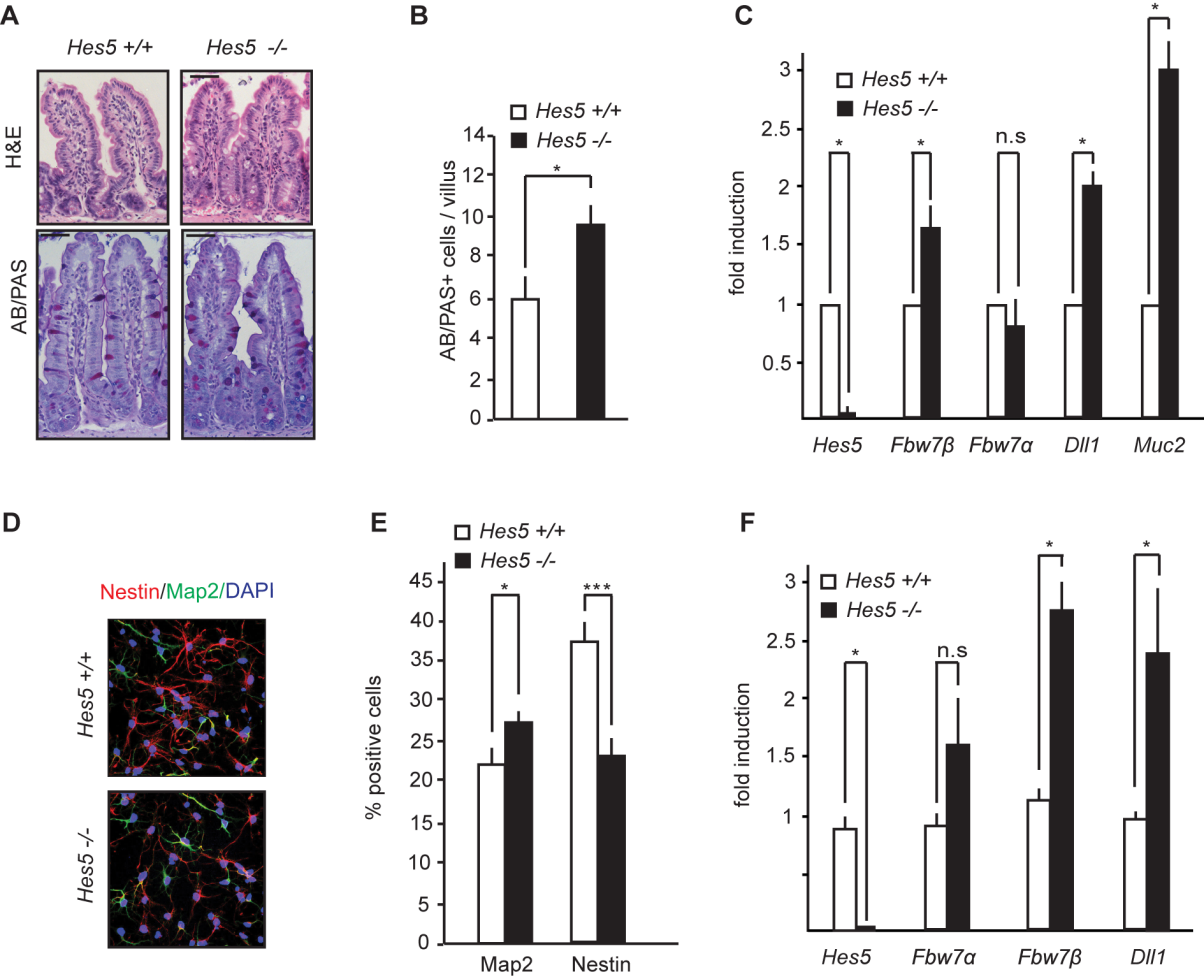
Increased goblet cell number and increased NSC differentiation in Hes5 KO mice. (a) H&E and AB/PAS staining in the intestinal tissue of Hes5^+/+^ or *Hes5* ^−/−^ mice. (b) Quantification of goblet (AB-PAS+) cells in the intestines of Hes5^+/+^ or *Hes5* ^−/−^ mice. (c) Q-PCR analysis of *Fbw7α*, *Fbw7β, Dll1*, *Hes5*, and *Muc2* in intestinal cells isolated from Hes5^+/+^ or *Hes5* ^−/−^ mice (relative fold induction after normalizing to actin ± SEM, *n*≥3 for each genotype). (d) Nestin, Map2, and DAPI staining on NSCs of Hes5^+/+^ or *Hes5* ^−/−^ mice, 3 d after differentiation. (e) Quantification of Nestin+ and Map2+ cells of Hes5^+/+^ or *Hes5* ^−/−^ NSCs, 3 d after differentiation (percentage positive cells ± SEM, *n*≥10 for each genotype). (f) Q-PCR analysis of *Fbw7α*, *Fbw7β, Dll1*, and *Hes5* from NSCs isolated from Hes5^+/+^ or *Hes5* ^−/−^ mice (relative fold induction after normalizing to actin ± SEM, *n*≥3 for each genotype).

To further investigate HES5 function in our proposed loop, we characterized the phenotype of *Hes5*-deficent mice in the intestine and NSCs. *Hes5* ^−/−^ mice are viable, but mutant phenotypes in various organ systems such as the eye, inner ear, and nervous system have been described [26]–[28]. However, the function of HES5 in the intestine and in NSCs has not been analysed. The absence of HES5 led to a significant increase in intestinal goblet cell number by approximately 50% (Figure 4a,b). Q-PCR analysis revealed increased *Fbw7β* expression, and also the mRNA levels of the HES target gene *Dll1* and the goblet cell marker *Muc2* were augmented while *Fbw7α* transcript levels remained unchanged (Figure 4c). Loss of HES5 in the brain caused no obvious phenotypic abnormalities, consistent with previous observations [29]. However, NSCs cultured from *Hes5* ^−/−^ animals showed significant premature differentiation of Nestin-positive cells with a concomitant mild increase of Map2 positive neurons (Figure 4d,e). Deletion of *Hes5* in NSCs also led to a significant increase in *Fbw7β* and *Dll1* expression (Figure 4f).

**Figure 5:**
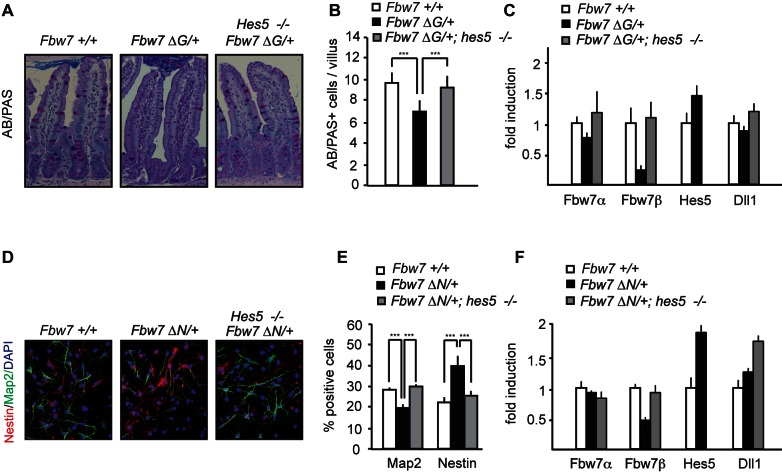
Hes5 deletion rescues Fbw7 haploinsufficiency in gut and NSCs. (a) H&E and AB/PAS staining in the intestines of *Fbw7^+/+^, Fbw7^ΔG/+^* or *Fbw7^ΔG/+^; Hes5* ^−/−^ mice. (b) Quantification of goblet (AB-PAS+) cells in the intestines of *Fbw7^+/+^, Fbw7^ΔG/+^*, or *Fbw7^ΔG/+^; Hes5* ^−/−^ mice. (c) Q-PCR analysis of *Fbw7α*, *Fbw7β, Hes5*, and *Dll1* in intestinal cells isolated from *Fbw7^+/+^, Fbw7^ΔG/+^* or *Fbw7^ΔG/+^; Hes5* ^−/−^ mice. (d) Nestin, Map2, and DAPI staining on NSCs isolated from *Fbw7^+/+^, Fbw7^ΔN/+^*, or *Fbw7^ΔN/+^; Hes5* ^−/−^ mice, 3 d after differentiation and (e) quantification of Nestin+ and Map2+ cells from these NSCs. (f) Q-PCR analysis of *Fbw7α*, *Fbw7β, Hes5*, and *Dll1* from NSCs isolated from *Fbw7^+/+^, Fbw7^ΔN/+^* or *Fbw7^ΔN/+^; Hes5* ^−/−^ mice. (percentage positive cells ± SEM, *n*≥10 for each genotype; relative fold induction after normalizing to actin ± SEM, *n*≥3 for each genotype).

We next tested whether the NICD1/HES5/FBW7β feedback loop might underlie the functional haploinsufficiency of FBW7. We generated compound mutant mice heterozygous for *Fbw7* in a *Hes5* ^−/−^ background (*Fbw7^ΔG/+^*; *Hes5* ^−/−^, *Fbw7^ΔN/+^*; *Hes5* ^−/−^ mice). Strikingly, goblet cell numbers were restored to wild-type levels in *Fbw7^ΔG/+^*; *Hes5* ^−/−^ mutant mice (Figure 5a,b), as were the numbers of Nestin-positive and Map2-positive cells in NSC differentiation cultures (Figure 5d,e). Importantly, the repression of *Fbw7*β transcription in heterozygotes was rescued in *Fbw7^ΔG/+^*; *Hes5* ^−/−^, and *Fbw7^ΔN/+^*; *Hes5* ^−/−^ mutant mice (Figure 5c,f). Thus, HES5 deficiency and FBW7 heterozygosity rescue each other, providing strong evidence that the two proteins are connected by a feedback loop.

### Mathematical Modelling of the Effects of the Fbw7 Feedback Loop in the Delta-Notch Lateral Inhibition Circuit

Our experiments imply that, overlaid on the standard gene regulatory circuit of Delta-Notch-mediated lateral inhibition, there is an intracellular feedback loop involving Fbw7: NICD stimulates expression of *Hes5*; Hes5 represses *Fbw7β*; and Fbw7β drives degradation of NICD. The net action of this NICD—>Hes5 —| Fbw7 —| NICD feedback loop is positive: it tends to amplify the effect of any change in any of the three components. This can explain why *Fbw7* is haploinsufficient, in the sense that loss of just one allele of the gene is enough to cause a marked shift in the ratio of secretory (low NICD) to absorptive (high NICD) cells in the gut, or of neurons to progenitors in the brain.

**Figure 6:**
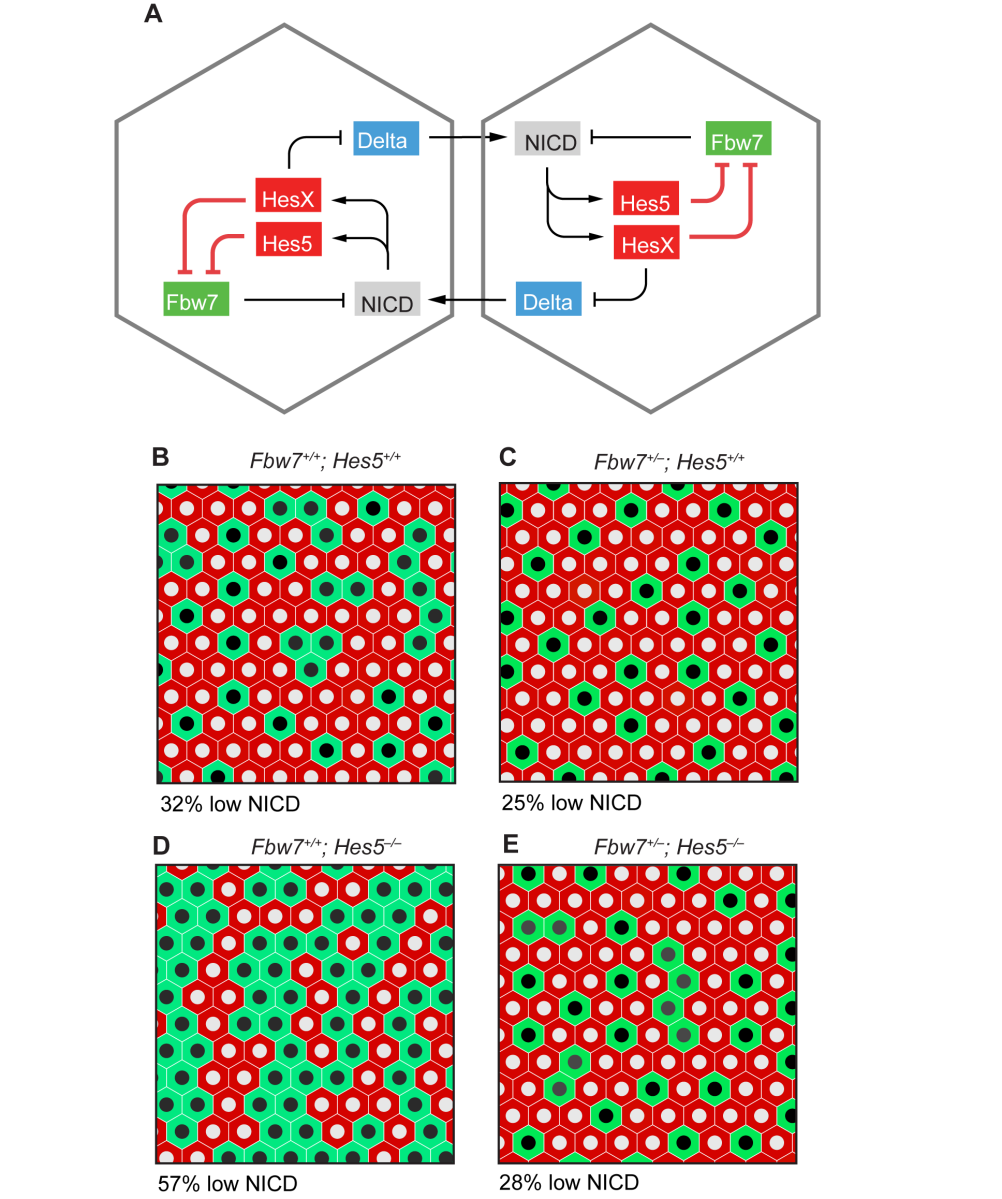
Mathematical modelling of the effects of the Fbw7 feedback loop in the Delta-Notch lateral inhibition circuit. (a) Diagram of the postulated gene regulatory network for a pair of adjacent cells. (b–e) Patterns of differentiation predicted by the mathematical model for an extended two-dimensional cell array; low-NICD cells are coloured green, high-NICD cells red. Four different genotypes are compared, showing the effect of halving the *Fbw7* gene dosage under conditions where *Hes5* is (*Hes5^+/+^*) or is not (*Hes5* ^−/−^) functional. The low-NICD cells correspond to secretory cells in the gut or to neurons in the brain. The percentage of low-NICD cells is shown below the picture of a typical pattern for each genotype. With the chosen model parameters, the predicted percentages of low-NICD cells for the four genotypes are in approximately the ratios observed in the gut (see Figures 4b and 5b). (In comparing the model with the real tissue, note that in the gut, the future absorptive cells continue dividing after the secretory cells have stopped [9], scaling up the observed numbers of absorptive cells relative to secretory cells in all genotypes.) See Materials and Methods and **Data S1** for details of the modelling.

Intuitive arguments are, however, untrustworthy when applied to systems with feedback. We have therefore investigated a mathematical model of the Delta-Notch lateral inhibition circuitry incorporating the intracellular Fbw7 feedback loop, to see whether it can indeed give rise to the observed phenomena. In Figure 6, we compare the predicted multicellular patterns of differentiation under four conditions, corresponding to the genotypes *Fbw7^+/+^;Hes5^+/+^* (b), *Fbw7^+/^* ^−^;*Hes^+/+^* (c), *Fbw7^+/+^;Hes5* ^−/−^ (d), and *Fbw7^+/^* ^−^;*Hes5* ^−/−^ (e). In (d), where Hes5 is absent, the proportion of secretory cells is increased; in (c), where Hes5 is present but one of the two *Fbw7* gene copies is defective, we see the opposite effect, reflecting haploinsufficiency of *Fbw7*; and in (e), where both types of mutation are present, their effects cancel out, restoring the normal ratio of secretory to absorptive cells. These results depend, of course, on the values assumed for the parameters in the model, for many of which we can only make rough guesses. The results of the modelling should therefore be viewed not so much as quantitative predictions, but rather as a demonstration that the experimental observations (Figures 1a, 4a, and 5a) are indeed consistent with a mechanism of the type proposed.

Intuitively, it seems that the Fbw7 loop superimposed on the standard lateral-inhibition circuitry should tend to amplify the differences between neighbouring cells and perhaps speed up the creation of a salt-and-pepper pattern. Moreover, as we have argued, it could explain why loss of a single Fbw7 gene copy has an unexpectedly large effect on the ratio of differentiated cell types in this final pattern.

## Discussion

Notch signalling is a key pathway that controls differentiation decisions in a vast number of cell types. SCF(Fbw7) is an important negative regulator of NICD function [30],[31], and many, though not all, of the phenotypes observed in *Fbw7* mutant animals can be attributed to deregulation of Notch activity [4],[5],[21],[32]–[34]. In this study we show that FBW7β is the isoform responsible for NICD degradation and also reveal that the functional relationship between FBW7β and Notch is not uni-directional, but that FBW7β and NICD are connected through a double-negative, i.e. positive, feedback loop.

We propose that the NICD/HES5/FBW7β feedback loop functions to refine the classical lateral inhibition mechanism (Figure 6a). Notch signalling represses transcription of Notch ligands, which leads to unequal levels of Notch signalling in neighbouring cells. We propose here that increasing levels of Notch activity results in reduced expression of *Fbw7β*, which in turn will lead to a further increase in NICD1 protein levels. Similarly, attenuation of Notch signalling will decrease NICD1 levels, as *Fbw7β* will be more highly expressed. Whereas NICD/Notch ligand regulation operates non-cell-autonomously, the NICD/HES5/FBW7β loop results in a cell-autonomous amplification of inequalities in Notch activity. This mechanism will help the cell to stably attain a Notch-high or Notch-low state, thereby solidifying cell fate decisions.

NICD1 stands out among all the SCF(Fbw7) substrates as it is the only substrate that is noticeably increased in Fbw7*^Δ/+^* heterozygous cells. Mechanistically, this is explained by the positive feedback causing repression of the wild-type *Fbw7* allele in *Fbw7^Δ/+^* heterozygous cells. Thus, instead of being reduced to just 50% of normal, *Fbw7* mRNA levels are reduced even further. Absolute quantification of *Fbw7* mRNA abundance in the intestine and NSCs has shown that the reduction in total *Fbw7* mRNA in heterozygous animals cannot be accounted for solely by the reduction in levels of the *Fbw7β* isoform. We believe that the small but consistent reduction of the more abundant *Fbw7α* mRNA (reflecting its moderate regulation by Hes5; see Figure 3a,b) contributes to the overall regulation of total *Fbw7* mRNA levels in heterozygous cells.

Previous reports have generated *Fbw7β*–specific knockout mice, which are viable, but comprehensive analyses of Notch-mediated phenotypes in brain or gut were not performed [35]. The same holds true for *Hes5* ^−/−^ mice, which had not been reported to have abnormalities in intestinal or NSC differentiation. In our analysis we have clearly shown that decreased levels of *Fbw7β* or loss of *Hes5* have a profound effect on patterns of differentiation in the intestine and in NSCs.

There are various reports regarding the localisation of FBW7β and its contribution to substrate turnover. Some accounts report that FBW7β localises to the cytosol [1],[36], whereas others have found it in the ER and Golgi [35]. Also in the cells we studied FBW7β localised predominantly in the cytoplasm but some nuclear localisation could also be observed, especially in response to proteasome inhibitor treatment (Figure S7a–c). Conversely FBW7β is able to interact with both endogenous NICD and overexpressed NICD (Figure S8a,b) and the ubiquitylation of overexpressed NICD is severely impaired in HCT116-*FBW7β*-*null* cells (Figure S8c). The cytoplasmic presence of FBW7β might even explain why the observed haploinsufficiency of *Fbw7^Δ/+^* animals is restricted to NICD1. Many Fbw7 substrates are predominantly nuclear, whereas NICD shuttles from the cytoplasm into the nucleus, and is thus present in both subcellular compartments. On a similar note, Ye et al. have shown that FBW7β is the predominant isoform responsible for CYCLIN-E turnover, which is primarily nuclear, but shuttles between cytoplasm and nucleus, like NICD [37].

A recent study, using homozygous isoform-specific *FBW7-null* mutations in human colon cancer HCT116 cells, has shown that FBW7*α* is the major isoform contributing to c-MYC and SREBP degradation [2]. We have used those cells to show that FBW7β is the isoform regulating NICD degradation. While our data suggest that *Fbw7β* is the major isoform regulating NICD degradation, *Fbw7α* possibly also contributes to the proposed feedback loop. Further, we can confirm previous studies showing that c-MYC is primarily degraded by FBW7α (Figure S6c) and [2]. The difference in substrate specificity and absolute abundance of the *Fbw7* isoforms, together with their heterogeneous tissue distribution, could also possibly explain the varying penetrance of *Fbw7* deletion in different organs.

*FBW7* is frequently mutated in a large variety of human tumours [24]. In particular, loss-of-function mutations in *FBW7* are very commonly found in human CRC [38]. Interestingly, about 70% of *FBW7* mutations are mono-allelic, and only about 30% of the colorectal tumours with *FBW7* mutations show loss-of-heterozygosity (LOH) [39],[40]. *FBW7* mRNA levels are significantly lower in human CRC tumour tissues than in normal intestinal tissue, and low *FBW7* expression correlates with poor prognosis [41]. In a mouse model for human CRC, it was clearly shown that *Hes5* expression was upregulated in tumours carrying *FBW7* heterozygous mutations when compared to tumours wild-type for *FBW7* [5]. Thus *FBW7* heterozygosity results in increased *Hes5* expression both in human colorectal tumours and in the *APC^min^;Fbw7^ΔG/+^* mouse model, suggesting that the NICD/HES5/FBW7β positive feedback loop is the molecular mechanism that underlies *FBW7* haploinsufficiency in tumour suppression.

Thus the feedback loop created through repression of *Fbw7β* by NICD plays a crucial part in Notch-regulated cell fate decisions, not only in normal tissues but also in the evolution of a large class of cancers.

## Materials and Methods

### Mouse Lines

*Fbw7* ^flox^, *Villin-cre, Nestin-cre*; APCmin/+ and Hes5^−/−^ mice have been described before [19],[20],[42]–[44].

### Cell Culture and Transfection

HCT116-wt, HCT116-Fbw7-α-null, and Fbw7-β-null cells have been described previously [2]. Cells were cultured in DMEM and 10% FBS. Cells were plated at subconfluence and transfected with Lipofectamine 2000 (Invitrogen).

NSCs were isolated as spheres from E13.5 fore- and midbrains of *Fbw7^f/f^*, *Fbw7^ΔN/+^*, *Fbw7^ΔN/+^*; *Hes5* ^−/−^ and *Hes5* ^−/−^ mouse embryos. Cells were initially cultured as spheres under self-renewal conditions, as previously described [4]. Adherent NSC cultures were derived as previously described [45] with minor modifications. Briefly, primary spheres were plated in Neurobasal Medium (Invitrogen) supplemented with 1% Penicillin/Streptomycin (10,000 U/ml; Invitrogen), 1% L-glutamine (200 mM; Invitrogen), 2% B27 supplement (Invitrogen), 1% N-2 supplement (Invitrogen), 20 ng/ml EGF (PeproTech), 20 ng/ml FGF-basic (PeproTech), and 1 µg/ml laminin (Sigma). All experiments were performed using adherent NSCs.

For differentiation, growth factors were withdrawn from the medium and 10% NeuroCult Differentiation Supplement (StemCell Technologies) was added. Under differentiation conditions, cells were plated on poly-L-ornithine (0.01% solution; Sigma; diluted 1∶10 in 150 mM disodium tetraborate; Sigma) coated cover slips.

For transfection, NSCs were plated at subconfluence and transfected with Lipofectamine 2000 according to the manufacturer's protocol (Invitrogen).

### Plasmids and Reagents

Cycloheximide was used at a final concentration of 100 µg/ml (Sigma). The Notch expression vector (Notch-IC-ΔOP) was a gift from Anna Bigas [46]. p-Super-sh-control, p-Super-sh-Hes5-1, and p-Super-sh-Hes5-2 were generated by cloning short hairpin containing oligos into pSuper vector following the manufacturer's instructions (Oligoengine). Silencing oligo sequences were sh-Hes5-1 (cagcctgcaccaggactac); sh-Hes5-2 (ggaagccggtggtggagaa). pCMV6-Hes5-gfp was purchased from Origene. pDest-flag was purchased from Invitrogen. pDest-Hes5-flag was generated by Gateway cloning of PCR-amplified Hes5 into pDest-flag. The oligonucleotide sequences used to amplify the DNA fragments for luciferase constructs are: pGL3-*Fbw7β*-cd fwd: 5′ TTTGACAGGGCATAGTCTCCTC 3′; pGL3-*Fbw7β*-cd rev: 5′ GCTCACAGTCTTTCCGTTATTATTTGC 3′; pGL3–*Fbw7β*-ef fwd: 5′ ATTGTCCCTGAAGGTAGTTGTG 3′; pGL3–*Fbw7β*-ef rev: 5′ TTTGGAGCCGACAGCATTTG 3′; pGL3–*Fbw7β*-cd^mut^: N box motif was modified via Geneart; pGL3–*Fbw7β*-ef^mut^: N box motif was modified via Geneart.

### Reporter Gene Assay

HCT116 cells were transfected with the indicated plasmids with Lipofectamine 2000 (Invitrogen). Transient transfections of the experimental samples and controls of Firefly and Renilla luciferase reporters was performed and measured using the Dual-Luciferase Reporter Assay System (Promega), 36 h posttransfection. Data are expressed as fold induction after being normalised using tk-renilla luciferase (mean ± SD; *n* = 3).

### ChIP

ChIP analysis was performed as described previously [47]. Cells were transfected with Lipofectamine 2000 (Invitrogen) with empty-flag or Hes5-flag prior to collection. Immunoprecipitations were carried out with anti-Flag antibody directly conjugated to agarose beads. The oligonucleotide sequences used to amplify the DNA fragments by q-PCR are: Fbw7α (ab)-fw: 5′-TGAATATCATGAAAAGATGCTGTATCAG-3′; Fbw7α (ab)-rev: 5′-TCAAGCATGTTTGCCTTTATGTTT-3′; Fbw7β (cd)-fw: 5′-TGGGCTTTTCTAGCTCAAGGAAT-3′; Fbw7β (cd)-rv: 5′-TTCATCTTGCAACTTCCTTCACA-3′; Fbw7β (ef)-fw: 5′-TCCCGAGAAGCGGTTTGAT-3′; Fbw7β (ef)-rv: 5′-GCAGAACCGGCAACAAAACT-3′; Ngn3-fw: 5′-CCCCTCCAGGACAGATGCT-3′; Ngn3-rv: 5′-CTGGTCAGGCCACCTCAGA -3′; Gapdh-fw: 5′-TGAGCAGTCCGGTGTCACTA-3′; Gapdh-rv: 5′-AAGAAGATGCGGCTGACTGT-3′; Actin-fw: 5′-GGATGCAGAAGGAGATCACTG-3′; Actin-rv: 5′-CGATCCACACGGAGTACTTG-3′; Cyclind1-fw: 5′-CGCCCCACCCCTCCAG-3′; Cyclind1-rv: 5′-CCGCCCAGACCCTCAGACT-3′.

Quantitative real-time PCR was accomplished with SYBR Green incorporation (Platinum Quantitative PCR SuperMix-UDG w/ROX, Invitrogen) using an ABI7900HT (Applied Bioscience), and the data were analyzed using the SDS 2.3 software.

### Quantitative RT-PCR

For qRT-PCR analysis, total mRNA was isolated from ileum fraction obtained as described before [5]. Total RNA was used from adherent NSC cultures. Results, normalized to β-actin, were presented as fold induction over control mice. The list of primers that were used for Q-PCR analysis of mouse tissues were: F-c-Jun: 5′-TGAAAGCTGTGTCCCCTGTC-3′; R-c-Jun: 5′-ATCACAGCACATGCCACTTC-3′; F-Fbw7α: 5′-CTGACCAGCTCTCCTCTCCATT-3′; R-Fbw7α: 5′-GCTGAACATGGTACAAGGCCA-3′; F-Fbw7β: 5′-TTGTCAGAGACTGCCAAGCAG-3′; R-Fbw7β: 5′-GACTTTGCATGGTTTCTTTCCC-3′; F-Fbw7 (exon5): 5′-TTCATTCCTGGAACCCAAAGA-3′; R-Fbw7 (exon5): 5′-TCCTCAGCCAAAATTCTCCAGTA-3′; F-Actin: 5′-TCTTTGCAGCTCCTTCGTTG-3′; R-Actin: 5′-ACGATGGAGGGGAATACAGC-3′; F-Hes1: 5′-TCAGCGAGTGCATGAACGA-3′; R-Hes1: 5′-TGCGCACCTCGGTGTTAAC-3′; F-Hes5: 5′-TGCAGGAGGCGGTACAGTTC-3′; R-Hes5: 5′-GCTGGAAGTGGTAAAGCAGCTT-3′; F-Dll1: 5′-CATGAACAACCTAGCCAATTGC-3′; R-Dll1: 5′-GCCCCAATGATGCTAACAGAA-3′; F-Muc2: 5′-TGTGGGACTTTTGCCATGTACT-3′; R-Muc2: 5′-GCAAGAGCACCTGTGATCCA-3′; F-c-Myc: 5′-CCTAGTGCTGCATGAGGAGA-3′; R-c-Myc: 5′-TCTTCCTCATCTTCTTGCTCTTC-3′.

### Western Blot Analysis

Immunoblots were carried out as previously described [4],[5]. Antibodies to c-JUN (BD biosciences), p-c-JUN^ser73^ (Cell Signalling), active-NOTCH-1 (Abcam), p-c-MYC (Cell Signaling), c-MYC (Santa Cruz), CYCLIN-E (Santa Cruz), and β-ACTIN (Sigma) were used.

### Histological Analysis

Mice were euthanized by cervical dislocation and the small intestines prepared for histology as described before [48]. Sections were cut at 4 µm for Haematoxylin & Eosin staining and PAS/AB staining. To quantify goblet cells, AB/PAS+ cells were quantified from at least 100 villi from comparable intestinal regions from at least 5 mice from each genotype and the data represented as the mean ± SEM.

### Intracellular Staining and FACS Analysis

HCT116 cells transfected with the indicated plasmids were fixed for 10 min in 1% PFA, permeabilized in PBS+0.5% Triton for 10 min at RT, and blocked in PBS+2% FCS for 30 min. After blocking, cells were incubated with anti-NICD antibody (1∶500 dilution in PBS+2% FCS) for 30 min. Cells were washed in PBS+2% FCS and incubated with donkey-anti-rabbit-Alexa647 secondary antibody (1∶1000 in PBS+2% FCS) for 30 min in the dark at RT. Cells were analysed in an LSRII cytometer. Overlay Histograms (Hes-GFP or sh-Hes5-GFP versus their controls) were represented as NICD-Alexa-647 versus cell numbers on GFP+ gated cells. The number of GFP+ cells quantified for each individual sample, the single histograms, and the percentage of cells in high-NICD and low-NICD state are indicated in Figure S5.

### Immunofluorescence

Cells from differentiation cultures were fixed for 20 min in 4% paraformaldehyde and permeabilized in ice-cold Methanol for 20 min. For immunocytochemistry, antibodies against NESTIN (BD (monoclonal)) and MAP2 (Sigma (monoclonal)) were used. DNA was counterstained with 4′-6-Diamidino-2-phenylindole (DAPI; Sigma).

### Mathematical Modelling

To describe the Delta-Notch-Fbw7-Hes gene regulatory circuit, we adapted a standard simple Delta-Notch lateral-inhibition model, adding the Fbw7 feedback loop as in Figure 6a. We represented the dynamics by a set of differential equations, which we solved numerically using Mathematica to determine the final state of a two-dimensional array of cells. The model assumes that there are two relevant *Hes* genes, *HesX* and *Hes5*, where *HesX* stands for one (or more) of the many other members of the Hes/Hey family that are expressed in gut and CNS. HesX (by itself) represses *Delta*, while HesX and Hes5 act in parallel to repress *Fbw7*. Loss of functional Hes5 thus leads roughly to a doubling of *Fbw7* expression and can be compensated by a halving of the *Fbw7* gene dosage. With our chosen model parameters, the Fbw7 positive feedback loop gives rise to bistability, allowing a cell exposed to a given level of Delta signalling from its neighbours (above a certain low Delta threshold) to exist in either a low- or a high-NICD state (as suggested by the data; see Figures 3c,d and S5a). This biases the outcome of Delta-Notch-mediated lateral inhibition. In the version of the model used to compute Figure 6, we postulate molecular lifetimes such that the dynamics of the Fbw7 loop are fast compared with the dynamics of the Delta-Notch loop. Each cell then moves rapidly to a low- or high-NICD state, with a relative probability dependent on the starting conditions and genotype, creating an initial random multicellular pattern that is subsequently adjusted by lateral inhibition. The adjustments follow a simple rule: thanks to bistability, low-NICD cells can persist regardless of the states of their neighbours, but any high-NICD cell that is entirely surrounded by other high-NICD cells is eventually converted to a low-NICD state. This is because high NICD entails a near-zero level of Delta production, and the high-NICD state becomes unstable when levels of Delta signalling from neighbours fall very low.

The model assumes that cells all start in an approximately similar state but with some small random variation from cell to cell, reflecting genetic noise, whose consequences are amplified through the Fbw7 and Delta-Notch feedback loops to give a final pepper-and-salt pattern. Results of the computation are shown for a 10×10 hexagonal array of cells, with cyclic boundary conditions.

Mathematical details of the model and values of the parameters are given in Data S1. The Mathematica program is available on request from julian.lewis@cancer.org.uk.

### Statistical Analysis

Statistical evaluation was performed by Student's unpaired *t* test. Data are presented as mean ± SEM. *p≤0.05 was considered statistically significant. **p≤0.01 was considered highly statistically significant. ***p≤0.001 was considered very highly statistically significant.

## Supporting Information

Data S1 Mathematical model.
(PDF)

Figure S1 NICD target gene analysis in Fbw7^ΔN/+^ and Fbw7^ΔG/+^ mice. (a) Quantification of NICD levels in intestine detected by Western blot in different experiments (pool of >3 mice each genotype per Western blot). Numbers represent fold induction over control after normalization to actin. (b) Quantification of NICD levels in NSCs detected by Western blot in different sets of mice (pool of >3 mice each genotype per Western blot). Numbers represent fold induction over control after normalization to actin. (c) Q-PCR analysis of *Hey1*, *Hey2*, *Hes1*, *Hes5*, *Hes6*, *Hes7*, *Jagged1*, *Jagged2*, *Dll1*, *Dll3*, and *Dll4* in wild-type or Fbw7^ΔN/+^ NSCs. (d) Q-PCR analysis of *Hey1*, *Hey2*, *Hes1*, *Hes5*, *Hes6*, *Hes7*, *Jagged1*, *Jagged2*, *Dll1*, *Dll3*, and *Dll4* in wild-type or Fbw7^ΔG/+^ intestinal tissue (ud, undetectable). (e) Q-PCR analysis of *Hes1* in wild-type, Fbw7^ΔG/+^ intestinal tissue and Fbw7^ΔN/+^ NSCs using four different sets of *Hes1* Q-PCR primers with specific sequences for the four different sets of *Hes1* Q-PCR primers used.
(TIF)

Figure S2 Absolute abundance of Fbw7*α* and Fbw7β mRNA in NSCs, Guts, and HCT116. Data presented in the table contain the calculated amount of molecules per microliter of *Fbw7α* and *Fbw7β* mRNA calculated as an extrapolation of the Ct values (from each sample) to the equation of the regression curve obtained using serial dilutions of *Fbw7α* or *Fbw7β* plasmids.
(TIF)

Figure S3 Endogenous HES5 chromatin IP analysis. ChIP was performed using HCT116 cells. HES5 binding to the consensus sites in *FBW7A*, *FBW7B*, and *NGN3* promoters was determined by Q-PCR. Data were represented as fold activation of percentage input versus IgG immunoprecipitated samples.
(TIF)

Figure S4 HES5 represses *Fbw7β* transcription. (a) Q-PCR analysis of *Fbw7α*, *Fbw7β*, *Hes5*, and *Hes1* in NSCs transfected with pcDNA3 or pcDNA3-NICD. (b) Q-PCR analysis of *Fbw7α*, *Fbw7β*, *Hes5*, and *Hes1* in NSCs transfected with p-Super-sh-control or p-Super-sh-Hes5-1 and p-Super-sh-Hes5-2 (specific silencers for Hes5).
(TIF)

Figure S5 FACS analysis of sh-Hes5 and Hes5-GFP transfected HCT116-*wt*, HCT116-*Fbw7α*-null, and HCT116-*Fbw7β*-null cells. (a) Single histograms displaying NICD-Alexa547 versus number of cells in sh-control-GFP/sh-Hes5-GFP transfected cells. (b) Percentage of NICD-low/NICD-high in sh-control-GFP/sh-Hes5-GFP transfected cells. (c) Table containing the number of GFP+ counted cells in each sample, the percentage of NICD-low/NICD-high cells, and the percentage increase in NICD-high cells of sh-Hes5-GFP transfected cells compared to sh-control-GFP transfected cells. (d) Single histograms displaying NICD versus number of cells in empty-GFP/Hes5-GFP transfected cells. (e) Percentage of NICD-low/NICD-high in empty-GFP/Hes5-GFP transfected cells. (f) Table containing the number of GFP+ counted cells in each sample, the percentage of NICD-low/NICD-high cells, and the percentage increase in NICD-high cells of Hes5-GFP transfected cells compared to empty-GFP transfected cells.
(TIF)

Figure S6 NICD target gene analysis in HCT116-*wt*, HCT116-*Fbw7α*-null and HCT116-*Fbw7β*-null cells. (a) FACS analysis of intracellular NICD in HCT116-*wt*, HCT116-*Fbw7α*-null, or HCT116-*Fbw7β*-null cells. (b) Q-PCR analysis of *Hey1*, *Hey2*, *Hes1*, *Hes5*, *Hes6*, *Jagged1*, *Jagged2*, *Dll1*, *Dll3*, and *Dll4* in HCT116-*wt*, HCT116-*Fbw7α*-null and HCT116-*Fbw7β*-null cells. (c) Western blot analysis of c-MYC and TUBULIN in HCT116-*wt*, HCT116-*Fbw7α*-null, or HCT116-*Fbw7β*-null cells after treatment with cyclohexamide for the indicated time points.
(TIF)

Figure S7 Subcellular localisation of Fbw7*β*. (a) Immunofluorescence of Hela cells transfected in the presence or absence of proteasome inhibitor (MG132) with pEGFP-C2-Fbw7*β*. (b) Immunoblot of nuclear and cytoplasmic extracts of 293T cells transfected with pEGFP-C2-Fbw7*β* in the presence of proteasome inhibitor (MG132) for GFP, LAMINB, and TUBULIN. (c) Immunoblot of nuclear and cytoplasmic extracts of HCT116 cells transfected with different concentrations of pCMV-Fbw7*β-flag* for Flag, LAMINB, and TUBULIN.
(TIF)

Figure S8 Fbw7*β* binds and ubiquitylates NICD. (a) HCT116-*wt* cells were transfected with Flag-tagged FBW7-alpha or FBW7-beta. Cell extracts were immunoprecipitated with anti-Flag and immunoblotted with anti-NICD. (b) HCT-Fbw7-*wt* cells were transfected with Flag-tagged FBW7-alpha ± Myc-tagged NICD or FBW7-beta ± Myc-tagged NICD. Cell extracts were immunoprecipitated with anti-Flag and immunoblotted with anti-MYC. (c) HCT116-*wt*, HCT116-*Fbw7α*-null, or HCT116-*Fbw7β*-null cells were transfected with Myc-tagged NICD and His-Ubiquitin. Ubiquitylated NICD was pulled down by Ni^2+^-NTA agarose beads and immunoblotted with anti MYC antibody.
(TIF)

Materials and Methods S1 Details of cell culture and transfections, IP assays, ubiquitylation assay, plasmids and reagents, and qRT-PCR used in supplementary figures.
(DOCX)

## Abbreviations

bHLH: basic helix-loop-helix
ChIP: chromatin IP
CRC: colorectal cancer
LOH: loss of heterozygocity
NICD: notch intracellular domain
NSC: neural stem cells
RGC: radial glial cell
SCF: Skp1-Cullin1-F-box
TA: transit-amplifying

## References

[1] Welcker M, Orian A, Grim JE, Eisenman RN, Clurman BE (2004) A nucleolar isoform of the Fbw7 ubiquitin ligase regulates c-Myc and cell size. Curr Biol 14: 1852–1857. 15498494 10.1016/j.cub.2004.09.083

[2] Grim JE, Gustafson MP, Hirata RK, Hagar AC, Swanger J, et al (2008) Isoform- and cell cycle-dependent substrate degradation by the Fbw7 ubiquitin ligase. J Cell Biol 181: 913–920. 18559665 10.1083/jcb.200802076 PMC2426948

[3] van Drogen F, Sangfelt O, Malyukova A, Matskova L, Yeh E, et al (2006) Ubiquitylation of cyclin E requires the sequential function of SCF complexes containing distinct hCdc4 isoforms. Mol Cell 23: 37–48. 16818231 10.1016/j.molcel.2006.05.020

[4] Hoeck JD, Jandke A, Blake SM, Nye E, Spencer-Dene B, et al (2010) Fbw7 controls neural stem cell differentiation and progenitor apoptosis via Notch and c-Jun. Nat Neurosci 13: 1365–1372. 20935640 10.1038/nn.2644

[5] Sancho R, Jandke A, Davis H, Diefenbacher ME, Tomlinson I, et al (2010) F-box and WD repeat domain-containing 7 regulates intestinal cell lineage commitment and is a haploinsufficient tumor suppressor. Gastroenterology 139: 929–941. 20638938 10.1053/j.gastro.2010.05.078

[6] Babaei-Jadidi R, Li N, Saadeddin A, Spencer-Dene B, Jandke A, et al (2011) FBXW7 influences murine intestinal homeostasis and cancer, targeting Notch, Jun, and DEK for degradation. J Exp Med 208: 295–312. 21282377 10.1084/jem.20100830 PMC3039859

[7] Scoville DH, Sato T, He XC, Li L (2008) Current view: intestinal stem cells and signaling. Gastroenterology 134: 849–864. 18325394 10.1053/j.gastro.2008.01.079

[8] Sancho E, Batlle E, Clevers H (2004) Signaling pathways in intestinal development and cancer. Annu Rev Cell Dev Biol 20: 695–723. 15473857 10.1146/annurev.cellbio.20.010403.092805

[9] Stamataki D, Holder M, Hodgetts C, Jeffery R, Nye E, et al (2011) Delta1 expression, cell cycle exit, and commitment to a specific secretory fate coincide within a few hours in the mouse intestinal stem cell system. PLoS One 6: e24484 doi:10.1371/journal.pone.0024484 21915337 10.1371/journal.pone.0024484 PMC3168508

[10] van Es JH, van Gijn ME, Riccio O, van den Born M, Vooijs M, et al (2005) Notch/gamma-secretase inhibition turns proliferative cells in intestinal crypts and adenomas into goblet cells. Nature 435: 959–963. 15959515 10.1038/nature03659

[11] Fre S, Huyghe M, Mourikis P, Robine S, Louvard D, et al (2005) Notch signals control the fate of immature progenitor cells in the intestine. Nature 435: 964–968. 15959516 10.1038/nature03589

[12] Gotz M, Barde YA (2005) Radial glial cells defined and major intermediates between embryonic stem cells and CNS neurons. Neuron 46: 369–372. 15882633 10.1016/j.neuron.2005.04.012

[13] Corbin JG, Gaiano N, Juliano SL, Poluch S, Stancik E, et al (2008) Regulation of neural progenitor cell development in the nervous system. J Neurochem 106: 2272–2287. 18819190 10.1111/j.1471-4159.2008.05522.x PMC2640107

[14] Yoon KJ, Koo BK, Im SK, Jeong HW, Ghim J, et al (2008) Mind bomb 1-expressing intermediate progenitors generate notch signaling to maintain radial glial cells. Neuron 58: 519–531. 18498734 10.1016/j.neuron.2008.03.018

[15] Gaiano N, Nye JS, Fishell G (2000) Radial glial identity is promoted by Notch1 signaling in the murine forebrain. Neuron 26: 395–404. 10839358 10.1016/s0896-6273(00)81172-1

[16] Borggrefe T, Oswald F (2009) The Notch signaling pathway: transcriptional regulation at Notch target genes. Cell Mol Life Sci 66: 1631–1646. 19165418 10.1007/s00018-009-8668-7 PMC11115614

[17] Fortini ME (2009) Notch signaling: the core pathway and its posttranslational regulation. Dev Cell 16: 633–647. 19460341 10.1016/j.devcel.2009.03.010

[18] Lewis J (1998) Notch signalling and the control of cell fate choices in vertebrates. Semin Cell Dev Biol 9: 583–589. 9892564 10.1006/scdb.1998.0266

[19] el Marjou F, Janssen KP, Chang BH, Li M, Hindie V, et al (2004) Tissue-specific and inducible Cre-mediated recombination in the gut epithelium. Genesis 39: 186–193. 15282745 10.1002/gene.20042

[20] Jandke A, Da Costa C, Sancho R, Nye E, Spencer-Dene B, et al (2011) The F-box protein Fbw7 is required for cerebellar development. Dev Biol 358: 201–212. 21827743 10.1016/j.ydbio.2011.07.030

[21] Tsunematsu R, Nakayama K, Oike Y, Nishiyama M, Ishida N, et al (2004) Mouse Fbw7/Sel-10/Cdc4 is required for notch degradation during vascular development. J Biol Chem 279: 9417–9423. 14672936 10.1074/jbc.M312337200

[22] Nateri AS, Riera-Sans L, Da Costa C, Behrens A (2004) The ubiquitin ligase SCFFbw7 antagonizes apoptotic JNK signaling. Science 303: 1374–1378. 14739463 10.1126/science.1092880

[23] Koepp DM, Schaefer LK, Ye X, Keyomarsi K, Chu C, et al (2001) Phosphorylation-dependent ubiquitination of cyclin E by the SCFFbw7 ubiquitin ligase. Science 294: 173–177. 11533444 10.1126/science.1065203

[24] Welcker M, Clurman BE (2008) FBW7 ubiquitin ligase: a tumour suppressor at the crossroads of cell division, growth and differentiation. Nat Rev Cancer 8: 83–93. 18094723 10.1038/nrc2290

[25] Jensen J, Pedersen EE, Galante P, Hald J, Heller RS, et al (2000) Control of endodermal endocrine development by Hes-1. Nat Genet 24: 36–44. 10615124 10.1038/71657

[26] Hojo M, Ohtsuka T, Hashimoto N, Gradwohl G, Guillemot F, et al (2000) Glial cell fate specification modulated by the bHLH gene Hes5 in mouse retina. Development 127: 2515–2522. 10821751 10.1242/dev.127.12.2515

[27] Ohtsuka T, Ishibashi M, Gradwohl G, Nakanishi S, Guillemot F, et al (1999) Hes1 and Hes5 as notch effectors in mammalian neuronal differentiation. EMBO J 18: 2196–2207. 10205173 10.1093/emboj/18.8.2196 PMC1171303

[28] Ohtsuka T, Sakamoto M, Guillemot F, Kageyama R (2001) Roles of the basic helix-loop-helix genes Hes1 and Hes5 in expansion of neural stem cells of the developing brain. J Biol Chem 276: 30467–30474. 11399758 10.1074/jbc.M102420200

[29] Hatakeyama J, Sakamoto S, Kageyama R (2006) Hes1 and Hes5 regulate the development of the cranial and spinal nerve systems. Dev Neurosci 28: 92–101. 16508307 10.1159/000090756

[30] Oberg C, Li J, Pauley A, Wolf E, Gurney M, et al (2001) The Notch intracellular domain is ubiquitinated and negatively regulated by the mammalian Sel-10 homolog. J Biol Chem 276: 35847–35853. 11461910 10.1074/jbc.M103992200

[31] Wu G, Lyapina S, Das I, Li J, Gurney M, et al (2001) SEL-10 is an inhibitor of notch signaling that targets notch for ubiquitin-mediated protein degradation. Mol Cell Biol 21: 7403–7415. 11585921 10.1128/MCB.21.21.7403-7415.2001 PMC99913

[32] Matsumoto A, Onoyama I, Sunabori T, Kageyama R, Okano H, et al (2011) Fbxw7-dependent degradation of Notch is required for control of “stemness” and neuronal-glial differentiation in neural stem cells. J Biol Chem 286: 13754–13764. 21349854 10.1074/jbc.M110.194936 PMC3075719

[33] Onoyama I, Suzuki A, Matsumoto A, Tomita K, Katagiri H, et al (2011) Fbxw7 regulates lipid metabolism and cell fate decisions in the mouse liver. J Clin Invest 121: 342–354. 21123947 10.1172/JCI40725 PMC3007132

[34] Tetzlaff MT, Yu W, Li M, Zhang P, Finegold M, et al (2004) Defective cardiovascular development and elevated cyclin E and Notch proteins in mice lacking the Fbw7 F-box protein. Proc Natl Acad Sci U S A 101: 3338–3345. 14766969 10.1073/pnas.0307875101 PMC373463

[35] Matsumoto A, Tateishi Y, Onoyama I, Okita Y, Nakayama K, et al (2011) Fbxw7beta resides in the endoplasmic reticulum membrane and protects cells from oxidative stress. Cancer Sci 102: 749–755. 21205095 10.1111/j.1349-7006.2011.01851.x

[36] Maruyama S, Hatakeyama S, Nakayama K, Ishida N, Kawakami K, et al (2001) Characterization of a mouse gene (Fbxw6) that encodes a homologue of Caenorhabditis elegans SEL-10. Genomics 78: 214–222. 11735228 10.1006/geno.2001.6658

[37] Ye X, Nalepa G, Welcker M, Kessler BM, Spooner E, et al (2004) Recognition of phosphodegron motifs in human cyclin E by the SCF(Fbw7) ubiquitin ligase. J Biol Chem 279: 50110–50119. 15364936 10.1074/jbc.M409226200

[38] Sjoblom T, Jones S, Wood LD, Parsons DW, Lin J, et al (2006) The consensus coding sequences of human breast and colorectal cancers. Science 314: 268–274. 16959974 10.1126/science.1133427

[39] Kemp Z, Rowan A, Chambers W, Wortham N, Halford S, et al (2005) CDC4 mutations occur in a subset of colorectal cancers but are not predicted to cause loss of function and are not associated with chromosomal instability. Cancer Res 65: 11361–11366. 16357143 10.1158/0008-5472.CAN-05-2565

[40] Miyaki M, Yamaguchi T, Iijima T, Takahashi K, Matsumoto H, et al (2009) Somatic mutations of the CDC4 (FBXW7) gene in hereditary colorectal tumors. Oncology 76: 430–434. 19420964 10.1159/000217811

[41] Iwatsuki M, Mimori K, Ishii H, Yokobori T, Takatsuno Y, et al (2010) Loss of FBXW7, a cell cycle regulating gene, in colorectal cancer: clinical significance. Int J Cancer 126: 1828–1837. 19739118 10.1002/ijc.24879

[42] Tronche F, Kellendonk C, Kretz O, Gass P, Anlag K, et al (1999) Disruption of the glucocorticoid receptor gene in the nervous system results in reduced anxiety. Nat Genet 23: 99–103. 10471508 10.1038/12703

[43] Moser AR, Pitot HC, Dove WF (1990) A dominant mutation that predisposes to multiple intestinal neoplasia in the mouse. Science 247: 322–324. 2296722 10.1126/science.2296722

[44] Cau E, Gradwohl G, Casarosa S, Kageyama R, Guillemot F (2000) Hes genes regulate sequential stages of neurogenesis in the olfactory epithelium. Development 127: 2323–2332. 10804175 10.1242/dev.127.11.2323

[45] Pollard SM, Conti L, Sun Y, Goffredo D, Smith A (2006) Adherent neural stem (NS) cells from fetal and adult forebrain. Cereb Cortex 16 Suppl 1: i112–i120. 16766697 10.1093/cercor/bhj167

[46] Bigas A, Martin DI, Milner LA (1998) Notch1 and Notch2 inhibit myeloid differentiation in response to different cytokines. Mol Cell Biol 18: 2324–2333. 9528802 10.1128/mcb.18.4.2324 PMC121486

[47] Aguilera C, Nakagawa K, Sancho R, Chakraborty A, Hendrich B, et al (2011) c-Jun N-terminal phosphorylation antagonises recruitment of the Mbd3/NuRD repressor complex. Nature 469: 231–235. 21196933 10.1038/nature09607

[48] Sancho R, Nateri AS, de Vinuesa AG, Aguilera C, Nye E, et al (2009) JNK signalling modulates intestinal homeostasis and tumourigenesis in mice. EMBO J 28: 1843–1854. 19521338 10.1038/emboj.2009.153 PMC2711188

