# Supplementary material for: Fbw7 Repression by Hes5 Creates a Feedback Loop That Modulates Notch-Mediated Intestinal and Neural Stem Cell Fate Decisions
Source: PLoS Biol. 2013 Jun 11;11(6):e1001586. doi: 10.1371/journal.pbio.1001586 (PMC3679002; doi:10.1371/journal.pbio.1001586)
Supplement: Data S1 — Mathematical model. (PDF) [file pbio.1001586.s001.pdf]

Supplementary Data S1: Mathematical model

Our model describes the dynamics of a Delta-Notch lateral inhibition system incorporating the Fbw7 feedback loop. A set of nine ordinary differential equations specifies how levels of the four mRNAs (*Delta*, *Hes1*, *Hes5*, and *Fbw7*) and five proteins (Delta, Hes1, Hes5, Fbw7, and NICD) forming the control system change with time in each cell as a function of the levels of those same molecules in the given cell and in its neighbours. To highlight the parameters that are critical and minimise the number that have to be specified, the model is formulated in terms of relative concentrations, with the unit of concentration for each mRNA and each protein defined to be equal to its maximal steady-state value (i.e. steady state value under conditions of maximal synthesis and/or minimal repression). With this non-dimensional system of concentration units, the equations reduce to the following simple form:

$$\frac{dD_m}{dt} = \mu_{D_m}\left(-D_m + \frac{1}{1+\left(\frac{H_{1p}}{K_{H1}}\right)^2}\right)$$

$$\frac{dD_p}{dt} = \mu_{D_p}\left(-D_p + D_m\right)$$

$$\frac{dH_{1m}}{dt} = \mu_{H_{1m}}\left(-H_{1m} + \frac{\left(\frac{N}{K_N}\right)^2}{1+\left(\frac{N}{K_N}\right)^2}\right)$$

$$\frac{dH_{1p}}{dt} = \mu_{H_{1p}}\left(-H_{1p} + H_{1m}\right)$$

$$\frac{dH_{5m}}{dt} = \mu_{H_{5m}}\left(-H_{5m} + \frac{\left(\frac{N}{K_N}\right)^2}{1+\left(\frac{N}{K_N}\right)^2}\right)$$

$$\frac{dH_{5p}}{dt} = \mu_{H_{5p}}\left(-H_{5p} + H_{5m}\right)$$

$$\frac{dF_m}{dt} = \mu_{F_m}\left(-F_m + \frac{1}{1+\left(\frac{H_{1p}}{K_{H1}}\right)^2+\left(\frac{H_{5p}}{K_{H5}}\right)^2}\right)$$

$$\frac{dF_p}{dt} = \mu_{F_p}\left(-F_p + F_m\right)$$

$$\frac{dN}{dt} = \mu_{N0}\left(-\left(1+\nu F_p\right)N + \frac{\left(\frac{\sum_{\text{neighbours}}\tilde{D}_p}{K_D}\right)^2}{1+\left(\frac{\sum_{\text{neighbours}}\tilde{D}_p}{K_D}\right)^2}\right)$$

Here, D, H_1, H_5, F and N stand for Delta, Hes1, Hes5, Fbw7 and NICD, respectively, with subscripts $_m$ and $_p$ distinguishing mRNA and protein. The coefficients μ are the degradation rates, i.e. the inverses of the molecular lifetimes. In each equation, the first (negative) term in the bracket on the right stands for degradation, the second (positive) term for synthesis. The stimulatory and repressive actions of Delta, Hes1, Hes5 and NICD are represented by Hill functions with Hill coefficient of 2, since evidence from other systems suggests that each of these molecules is likely to act as a dimer.

For NICD, the basal degradation rate is μ_{N0}, while the additional contribution to degradation due to Fbw7 is taken to be simply proportional to the Fbw7 concentration, with coefficient v, so that at the maximal (i.e. unit) concentration of Fbw7, the degradation of NICD is speeded up by a factor $1+v$. The rate of production of NICD is an increasing function of the sum of the levels of Delta protein in the neighbouring cells, denoted $\check{D}_p$.

Calculations shown in Figure 6 were done for a 10x10 hexagonally packed sheet of cells, with cyclic boundary conditions and a starting condition in which all the genes are expressed but at randomly variable low levels. The values of the parameters used in the computation of Figure 6 are specified in the Table below.

**parameter**	**significance**	**value**	**units**
μ_{Dm}	degradation rate	0.003	min^{-1}
μ_{Dp}	degradation rate	0.1	min^{-1}
μ_{Fm}	degradation rate	0.1	min^{-1}
μ_{Fp}	degradation rate	0.1	min^{-1}
μ_{H1m}	degradation rate	0.1	min^{-1}
μ_{H1p}	degradation rate	0.1	min^{-1}
μ_{H5m}	degradation rate	0.1	min^{-1}
μ_{H5p}	degradation rate	0.1	min^{-1}
μ_{N0}	degradation rate	0.01	min^{-1}
v	$1+v$ = factor by which maximal (unit) concentration of Fbw7 increases the NICD degradation rate	50	non-dimensional
K_D	critical concn/max concn	0.1	non-dimensional
K_{H1}	critical concn/max concn	0.01	non-dimensional
K_{H5}	critical concn/max concn	0.01	non-dimensional
K_N	critical concn/max concn	1	non-dimensional
initial D_m/max D_m	starting condition	0.5±0.5	non-dimensional

initial D_p/max D_p	starting condition	0.5±0.5	non-dimensional
initial H_{1m}/max H_{1m}	starting condition	0.05±0.05	non-dimensional
initial H_{1p}/max H_{1p}	starting condition	0.05±0.05	non-dimensional
initial H_{5m}/max H_{5m}	starting condition	0.05±0.05	non-dimensional
initial H_{5p}/max H_{5p}	starting condition	0.05±0.05	non-dimensional
initial F_m/max F_m	starting condition	0.5±0.3	non-dimensional
initial F_p/max F_p	starting condition	0.5±0.3	non-dimensional
initial N/max N	starting condition	0.5±0.5	non-dimensional
