# Supplementary figures and images for: Fbw7 Repression by Hes5 Creates a Feedback Loop That Modulates Notch-Mediated Intestinal and Neural Stem Cell Fate Decisions
Source: PLoS Biol. 2013 Jun 11;11(6):e1001586. doi: 10.1371/journal.pbio.1001586 (PMC3679002; doi:10.1371/journal.pbio.1001586)

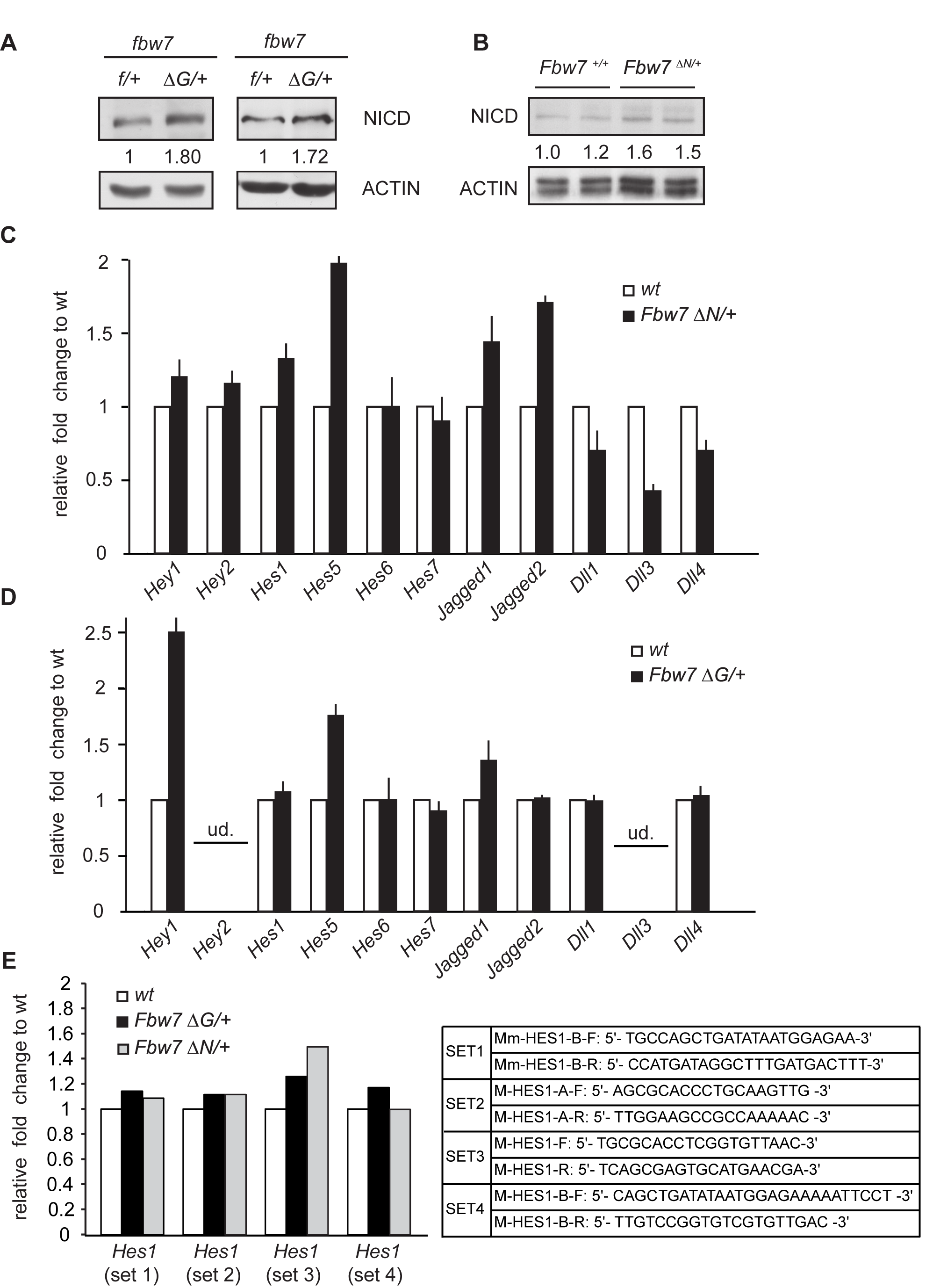

Supplement: Figure S1 — NICD target gene analysis in Fbw7ΔN/+ and Fbw7ΔG/+ mice. (a) Quantification of NICD levels in intestine detected by Western blot in different experiments (pool of >3 mice each genotype per Western blot). Numbers represent fold induction over control after normalization to actin. (b) Quantification of NICD levels in NSCs detected by Western blot in different sets of mice (pool of >3 mice each genotype per Western blot). Numbers represent fold induction over control after normalization to actin. (c) Q-PCR analysis of Hey1, Hey2, Hes1, Hes5, Hes6, Hes7, Jagged1, Jagged2, Dll1, Dll3, and Dll4 in wild-type or Fbw7ΔN/+ NSCs. (d) Q-PCR analysis of Hey1, Hey2, Hes1, Hes5, Hes6, Hes7, Jagged1, Jagged2, Dll1, Dll3, and Dll4 in wild-type or Fbw7ΔG/+ intestinal tissue (ud, undetectable). (e) Q-PCR analysis of Hes1 in wild-type, Fbw7ΔG/+ intestinal tissue and Fbw7ΔN/+ NSCs using four different sets of Hes1 Q-PCR primers with specific sequences for the four different sets of Hes1 Q-PCR primers used. (TIF) [file pbio.1001586.s002.tif]

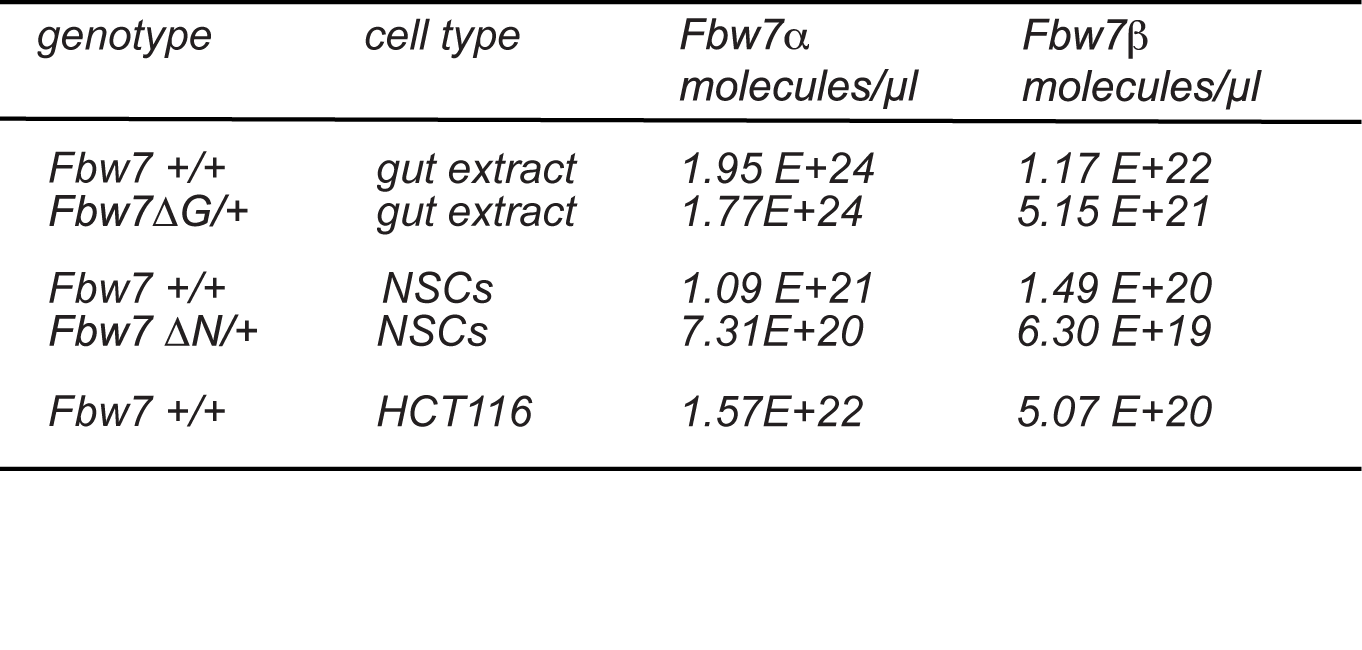

Supplement: Figure S2 — Absolute abundance of Fbw7α and Fbw7β mRNA in NSCs, Guts, and HCT116. Data presented in the table contain the calculated amount of molecules per microliter of Fbw7α and Fbw7β mRNA calculated as an extrapolation of the Ct values (from each sample) to the equation of the regression curve obtained using serial dilutions of Fbw7α or Fbw7β plasmids. (TIF) [file pbio.1001586.s003.tif]

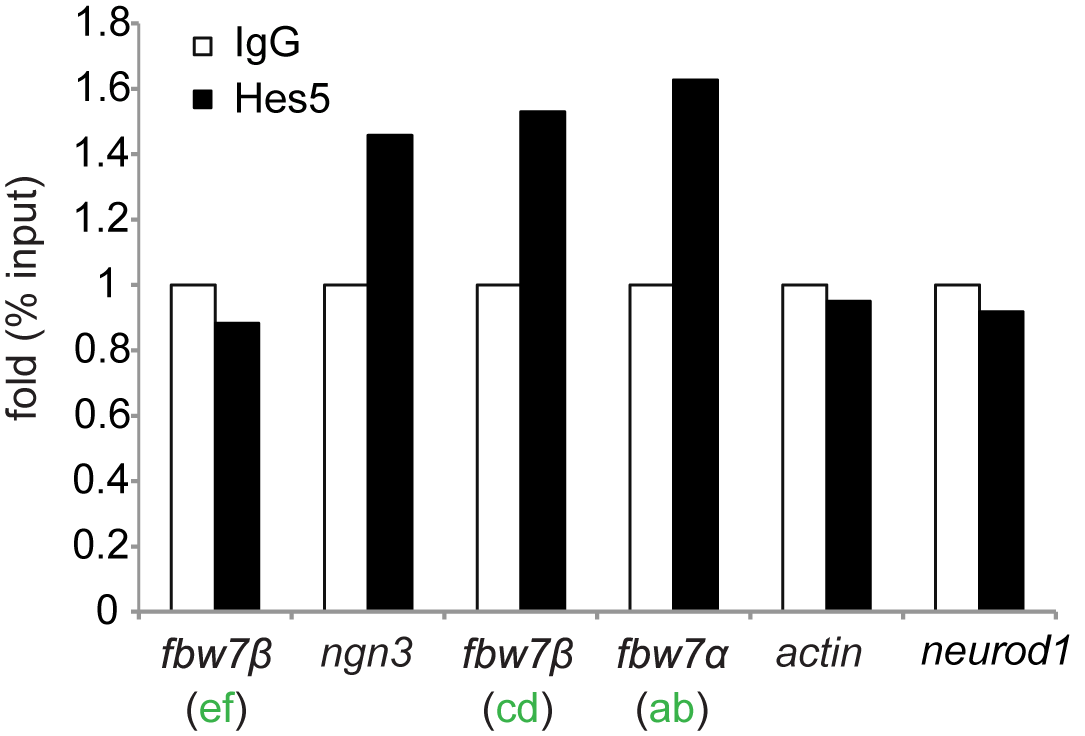

Supplement: Figure S3 — Endogenous HES5 chromatin IP analysis. ChIP was performed using HCT116 cells. HES5 binding to the consensus sites in FBW7A, FBW7B, and NGN3 promoters was determined by Q-PCR. Data were represented as fold activation of percentage input versus IgG immunoprecipitated samples. (TIF) [file pbio.1001586.s004.tif]

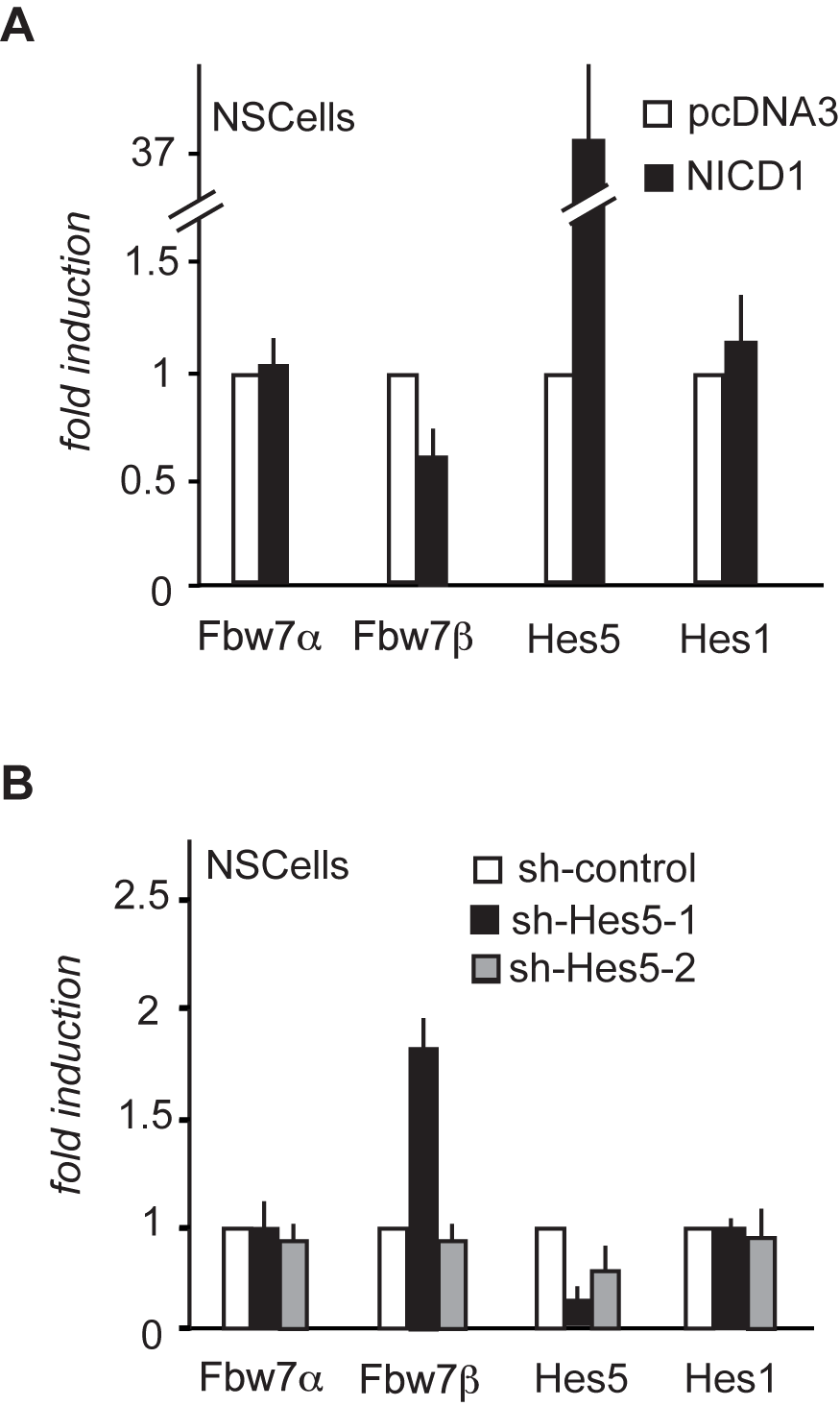

Supplement: Figure S4 — HES5 represses Fbw7β transcription. (a) Q-PCR analysis of Fbw7α, Fbw7β, Hes5, and Hes1 in NSCs transfected with pcDNA3 or pcDNA3-NICD. (b) Q-PCR analysis of Fbw7α, Fbw7β, Hes5, and Hes1 in NSCs transfected with p-Super-sh-control or p-Super-sh-Hes5-1 and p-Super-sh-Hes5-2 (specific silencers for Hes5). (TIF) [file pbio.1001586.s005.tif]

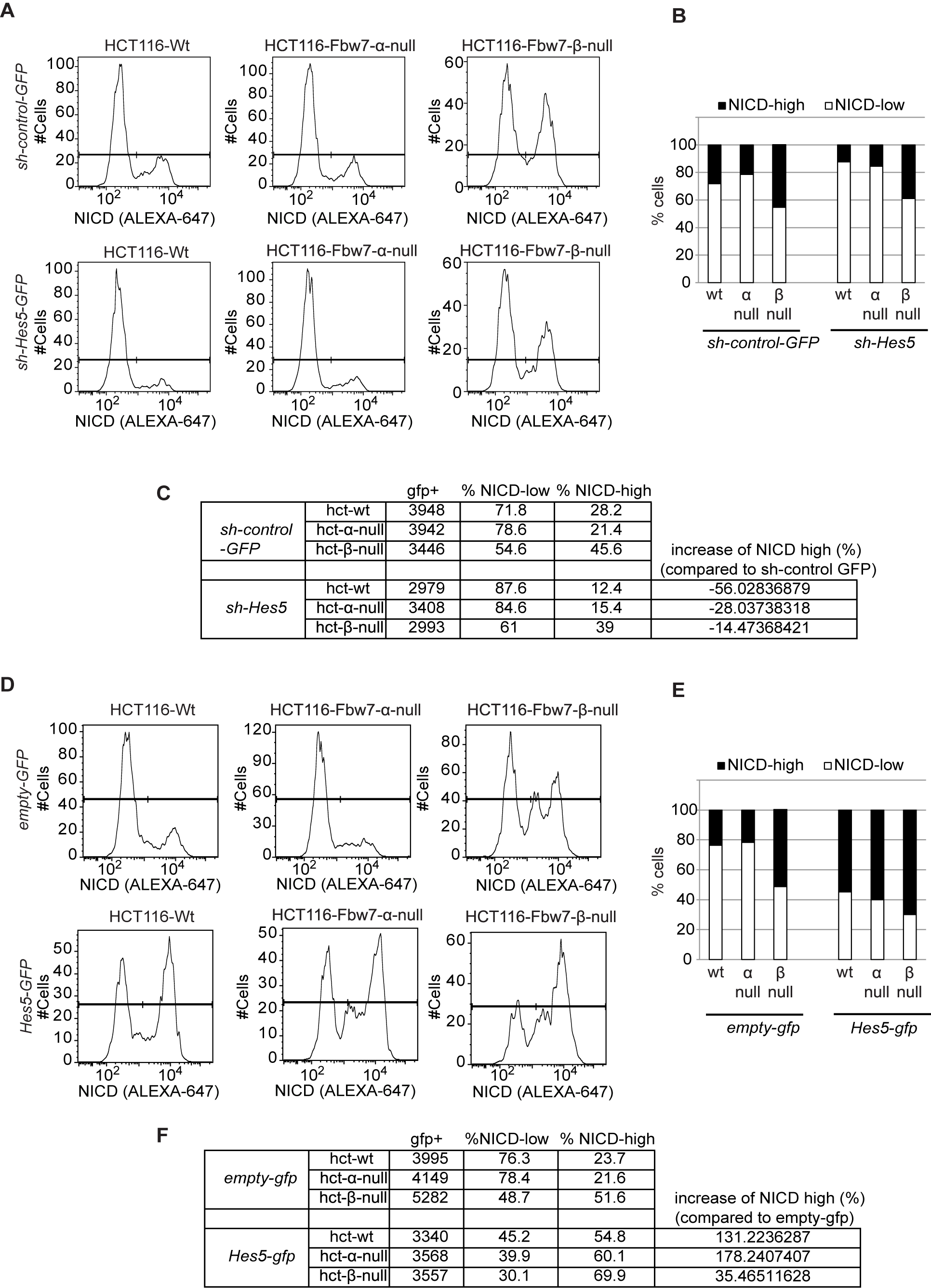

Supplement: Figure S5 — FACS analysis of sh-Hes5 and Hes5-GFP transfected HCT116-wt, HCT116-Fbw7α-null, and HCT116-Fbw7β-null cells. (a) Single histograms displaying NICD-Alexa547 versus number of cells in sh-control-GFP/sh-Hes5-GFP transfected cells. (b) Percentage of NICD-low/NICD-high in sh-control-GFP/sh-Hes5-GFP transfected cells. (c) Table containing the number of GFP+ counted cells in each sample, the percentage of NICD-low/NICD-high cells, and the percentage increase in NICD-high cells of sh-Hes5-GFP transfected cells compared to sh-control-GFP transfected cells. (d) Single histograms displaying NICD versus number of cells in empty-GFP/Hes5-GFP transfected cells. (e) Percentage of NICD-low/NICD-high in empty-GFP/Hes5-GFP transfected cells. (f) Table containing the number of GFP+ counted cells in each sample, the percentage of NICD-low/NICD-high cells, and the percentage increase in NICD-high cells of Hes5-GFP transfected cells compared to empty-GFP transfected cells. (TIF) [file pbio.1001586.s006.tif]

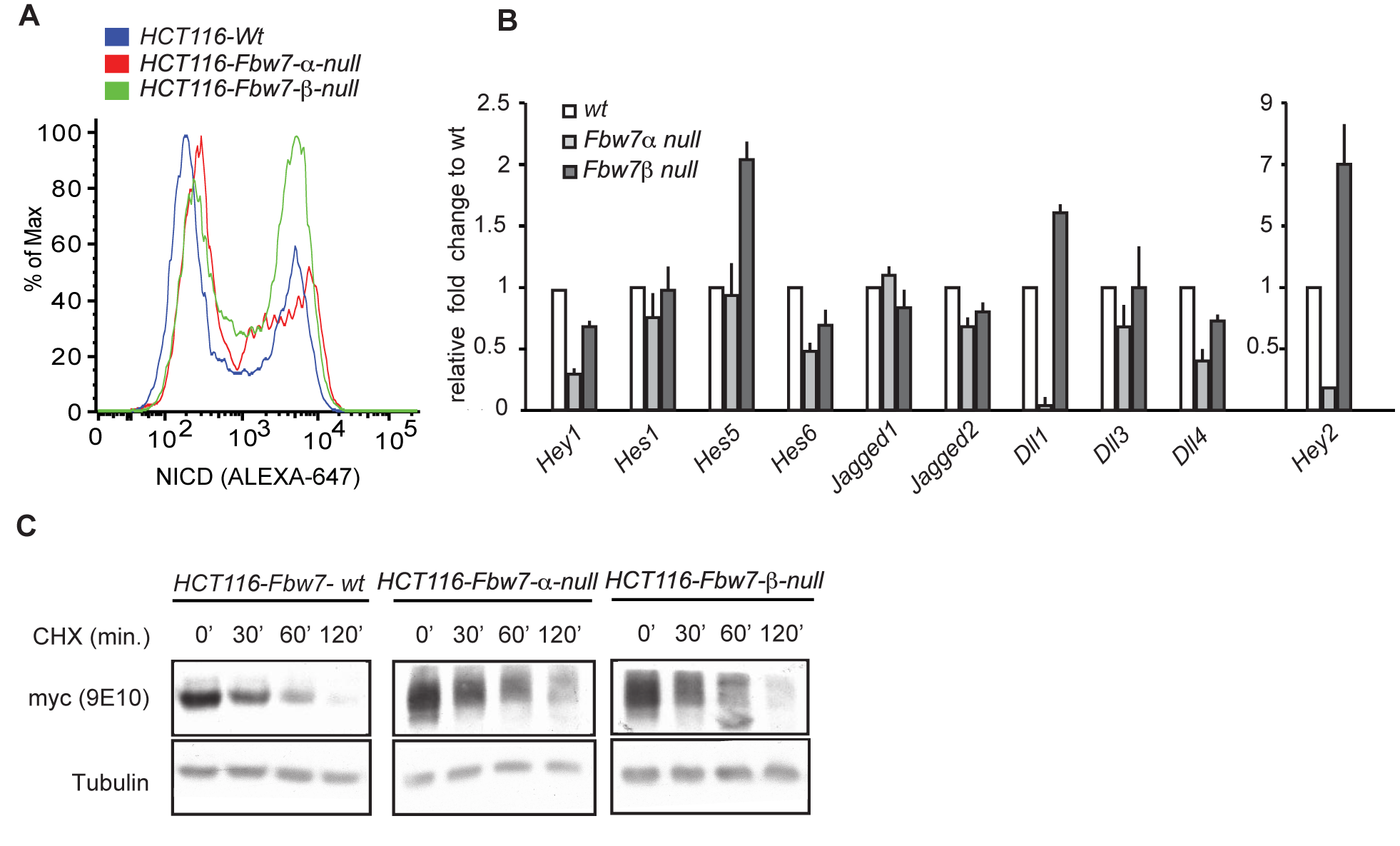

Supplement: Figure S6 — NICD target gene analysis in HCT116-wt, HCT116-Fbw7α-null and HCT116-Fbw7β-null cells. (a) FACS analysis of intracellular NICD in HCT116-wt, HCT116-Fbw7α-null, or HCT116-Fbw7β-null cells. (b) Q-PCR analysis of Hey1, Hey2, Hes1, Hes5, Hes6, Jagged1, Jagged2, Dll1, Dll3, and Dll4 in HCT116-wt, HCT116-Fbw7α-null and HCT116-Fbw7β-null cells. (c) Western blot analysis of c-MYC and TUBULIN in HCT116-wt, HCT116-Fbw7α-null, or HCT116-Fbw7β-null cells after treatment with cyclohexamide for the indicated time points. (TIF) [file pbio.1001586.s007.tif]

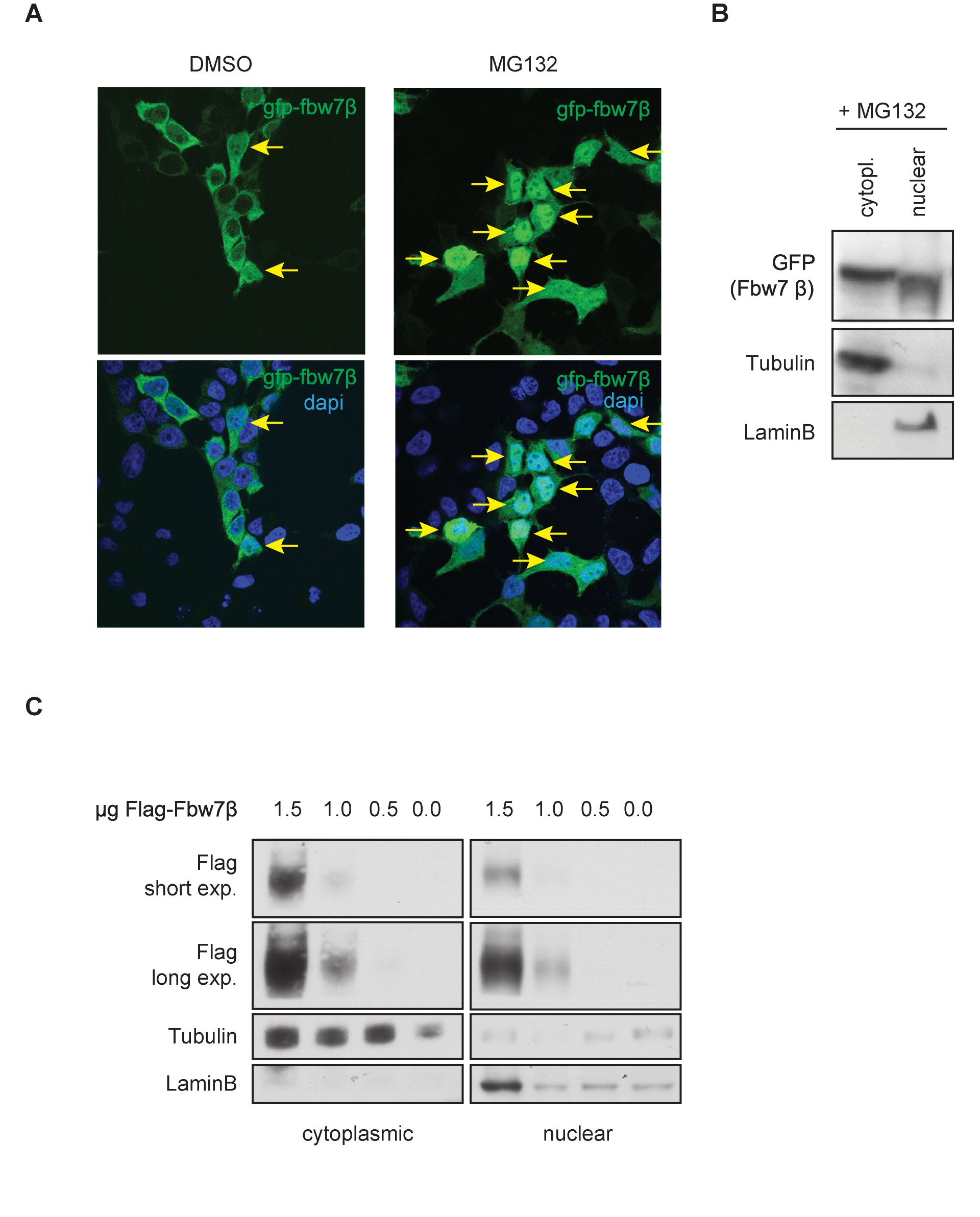

Supplement: Figure S7 — Subcellular localisation of Fbw7β. (a) Immunofluorescence of Hela cells transfected in the presence or absence of proteasome inhibitor (MG132) with pEGFP-C2-Fbw7β. (b) Immunoblot of nuclear and cytoplasmic extracts of 293T cells transfected with pEGFP-C2-Fbw7β in the presence of proteasome inhibitor (MG132) for GFP, LAMINB, and TUBULIN. (c) Immunoblot of nuclear and cytoplasmic extracts of HCT116 cells transfected with different concentrations of pCMV-Fbw7β-flag for Flag, LAMINB, and TUBULIN. (TIF) [file pbio.1001586.s008.tif]

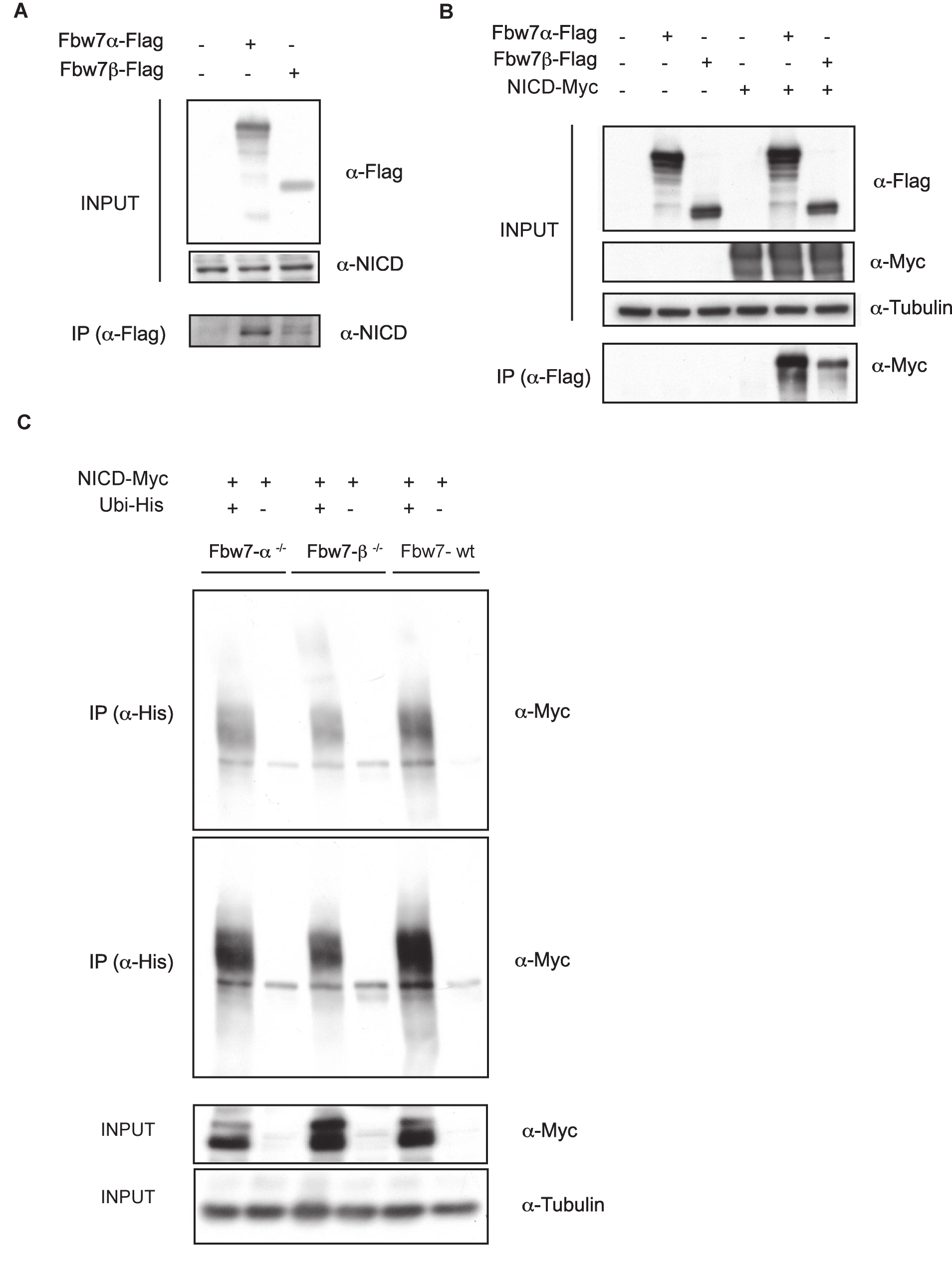

Supplement: Figure S8 — Fbw7β binds and ubiquitylates NICD. (a) HCT116-wt cells were transfected with Flag-tagged FBW7-alpha or FBW7-beta. Cell extracts were immunoprecipitated with anti-Flag and immunoblotted with anti-NICD. (b) HCT-Fbw7-wt cells were transfected with Flag-tagged FBW7-alpha ± Myc-tagged NICD or FBW7-beta ± Myc-tagged NICD. Cell extracts were immunoprecipitated with anti-Flag and immunoblotted with anti-MYC. (c) HCT116-wt, HCT116-Fbw7α-null, or HCT116-Fbw7β-null cells were transfected with Myc-tagged NICD and His-Ubiquitin. Ubiquitylated NICD was pulled down by Ni2+-NTA agarose beads and immunoblotted with anti MYC antibody. (TIF) [file pbio.1001586.s009.tif]
