# Supplementary material for: Fbw7 Repression by Hes5 Creates a Feedback Loop That Modulates Notch-Mediated Intestinal and Neural Stem Cell Fate Decisions
Source: PLoS Biol. 2013 Jun 11;11(6):e1001586. doi: 10.1371/journal.pbio.1001586 (PMC3679002; doi:10.1371/journal.pbio.1001586)
Supplement: Materials and Methods S1 — Details of cell culture and transfections, IP assays, ubiquitylation assay, plasmids and reagents, and qRT-PCR used in supplementary figures. (DOCX) [file pbio.1001586.s010.docx]

**SUPPORTING MATERIALS AND METHODS**

**Cell Culture and Transfection.**

293T, HCT116 and Hela cells were cultured in DMEM and 10% FBS. Cells were plated at subconfluence and transfected with Lipofectamine 2000 ([Invitrogen](http://www.pnas.org/cgi/redirect-inline?ad=Invitrogen)). Proteasome inhibitor (MG132, Merck) treatment was performed 3hrs prior to cell harvest. Nuclear and cytoplasmic fractionation was performed as previously described [[49](#_ENREF_49)].

**Immunoprecipitation assay.**

Cells were transfected with the indicated plasmids. 24h after transfection cells were treated with proteasome inhibitor (MG132) for 5h. After proteasome inhibitor treatment, cells were washed with cold phosphate-buffered saline (PBS) and subsequently resuspended in NP40 Lysis buffer (150 mM NaCl, 80 mM Tris-HCl (pH 7.2), 0.2% NP-40, 10% glycerol) supplemented with complete protease inhibitor cocktail (Sigma). The resuspended pellet was sonicated for 10 sec and lysates were cleared by centrifugation for 15 min at 4°C. Lysates were immunoprecipitated for Flag using anti-Flag conjugated agarose for 2 hrs by rotation at 4°C. Immunoprecipitates were washed five times with lysis buffer, and sample proteins were separated by SDS-PAGE and subsequently transferred onto Nitrocellulose membranes and blotted with the indicated antibodies.

**Ubiquitylation assay.**

Cells were transfected with the indicated plasmids. 24h after transfection cells were treated with proteasome inhibitor (MG132) for 3hrs. After proteasome inhibitor treatment, cells were washed with cold phosphate-buffered saline (PBS) and subsequently subjected to *in vivo* ubiquitylation assays. His–Ubiquitin was affinity purified with nickel-NTA-agarose beads, as described previously [[49](#_ENREF_49)], and sample proteins were separated by SDS-PAGE and subsequently transferred onto Nitrocellulose membranes and blotted with the indicated antibodies.

**Plasmids and Reagents**

LAMIN B antibody was purchased from Santa Cruz, β-ACTIN and GFP (rabbit) from Abcam, anti-c-MYC (rabbit) from Santa Cruz, anti- TUBULIN (mouse) from Chemicon and anti-HES5 (rabbit) from Millipore. The NICD overexpression plasmid (pC52-mNICD-MT) used in the ubiquitylation assay and the Co-IP has been reported previously [2]. pEGFP-C2-Fbw7*β* was generated by cloning mouse Fbw7*β into* pEGFP-C2 (Clonetech) using EcoRI and SalI restriction sites

**Quantitative RT-PCR**

cDNA was prepared as described in main Material and Methods. Serial dilutions of plasmid DNA in RNA were used as a standard curve to determine absolute Fbw7 isoform levels in HCT116 cells, intestinal extracts and NSCs (displayed as molecules/µl).

The list of primers that were used for Q-PCR analysis of mouse tissues were:

F-Hey1: 5’- CATGAAGAGAGCTCACCCAGA-3’

R-Hey1: 5’- CGCCGAACTCAAGTTTCC-3’

F-Hey2: 5’- ATTGCAAATGACAGTGGATCAT-3’

R-Hey2: 5’- AGCATGGGCATCAAAGTAGC-3’

F-Hes6: 5'- GGCCAGGAGGATGAGGAC-3'

R-Hes6: 5'- GCTCCTGAAGACTCTCGTTGA -3'

F-Hes7: 5'- CGGAGGAGCAATGGTCAC-3'

R-Hes7: 5'- TTCTAGGCTGCGGTTGATG -3'

F-Jag1: 5’- ATTGCAAATGACAGTGGATCAT-3’

R-Jag1: 5’- ATCGATGTTTGTGGAACACG-3’

F-Jag2: 5'- CAATGACACCACTCCAGATGAG -3'

R-Jag2: 5'- GTTCTCATCACAGCGCACTC -3'

The list of primers that were used for Q-PCR analysis of human HCT116 cells were:

F-Hey1: 5’- CTCAGTGGCCTCCCTGTC-3’

R-Hey1: 5’- GCTCAGTGCATTGGGAGAC-3’

F-Hey2: 5’- TTTGAAGATGCTTCAGGCAA-3’

R-Hey2: 5’- GGCACTCTCGGAATCCTATG-3’

F-Hes6: 5'- AAGCTGGAGAACGCCGA-3'

R-Hes6: 5'- ACTGGATGTAGCCGGCAG-3'

F-Dll1: 5'- CTTCCCCTTCGGCTTCAC-3'

R-Dll1: 5'- GGGTTTTCTGTTGCGAGGT-3'

F-Dll3: 5'- CAACTGTGAGAAGAGGGTGGA-3'

R-Dll3: 5'- CCAGGTCCAGGCAGAGTC-3'

F-Dll4: 5'- ATGCAAGAATGGGGCAAC-3'

R-Dll4: 5'- CGACAGGTGCAGGTGTAGC-3'

F-Jag1: 5’- CACAGTGGTGCCAAGTGC-3’

R-Jag1: 5’- GCCCCATCTGGTATCACACT-3’

F-Jag2: 5'- TCATCCCCTTCCAGTTCG-3'

R-Jag2: 5'- ATGCGACACTCGCTCGAT-3'
